# Programmed DNA elimination of germline development genes in songbirds

**DOI:** 10.1038/s41467-019-13427-4

**Published:** 2019-11-29

**Authors:** Cormac M. Kinsella, Francisco J. Ruiz-Ruano, Anne-Marie Dion-Côté, Alexander J. Charles, Toni I. Gossmann, Josefa Cabrero, Dennis Kappei, Nicola Hemmings, Mirre J. P. Simons, Juan Pedro M. Camacho, Wolfgang Forstmeier, Alexander Suh

**Affiliations:** 1 Department of Ecology and Genetics – Evolutionary Biology, Evolutionary Biology Centre (EBC), Science for Life Laboratory, Uppsala University, SE-752 36 Uppsala, Sweden; 2 Department of Genetics, University of Granada, E-18071 Granada, Spain; 3 Department of Molecular Biology & Genetics, Cornell University, Ithaca, NY 14853 USA; 4 Department of Animal and Plant Sciences, University of Sheffield, S10 2TN Sheffield, UK; 5 Cancer Science Institute of Singapore, National University of Singapore, 117599 Singapore, Singapore; 6 Department of Biochemistry, Yong Loo Lin School of Medicine, National University of Singapore, 117596 Singapore, Singapore; 7 Max Planck Institute for Ornithology, D-82319 Seewiesen, Germany; 8 Present Address: Laboratory of Experimental Virology, Department of Medical Microbiology, Amsterdam UMC, University of Amsterdam, 1105 AZ Amsterdam, The Netherlands; 9 Present Address: Department of Organismal Biology – Systematic Biology, Evolutionary Biology Centre (EBC), Science for Life Laboratory, Uppsala University, SE-752 36 Uppsala, Sweden; 10 Present Address: Département de Biologie, Université de Moncton, Moncton, NB E1A 3E9 Canada; 11 Present Address: Department of Animal Behaviour, Bielefeld University, D-33501 Bielefeld, Germany

**Keywords:** Germline development, Evolutionary genetics, Molecular evolution, Genome evolution

## Abstract

In some eukaryotes, germline and somatic genomes differ dramatically in their composition. Here we characterise a major germline–soma dissimilarity caused by a germline-restricted chromosome (GRC) in songbirds. We show that the zebra finch GRC contains >115 genes paralogous to single-copy genes on 18 autosomes and the Z chromosome, and is enriched in genes involved in female gonad development. Many genes are likely functional, evidenced by expression in testes and ovaries at the RNA and protein level. Using comparative genomics, we show that genes have been added to the GRC over millions of years of evolution, with embryonic development genes *bicc1* and *trim71* dating to the ancestor of songbirds and dozens of other genes added very recently. The somatic elimination of this evolutionarily dynamic chromosome in songbirds implies a unique mechanism to minimise genetic conflict between germline and soma, relevant to antagonistic pleiotropy, an evolutionary process underlying ageing and sexual traits.

## Abstract (web summary)

Songbirds have extensive germline–soma genome differences due to developmental elimination of a germline-specific chromosome (GRC). Here, the authors show that the GRC contains dozens of expressed developmental genes, some of which have been on the GRC since the ancestor of all songbirds.

## Introduction

Not all cells of an organism must contain the same genome. Dramatic differences between germline and somatic genomes can occur by programmed DNA elimination of chromosomes or fragments thereof. This phenomenon happens during the germline–soma differentiation of ciliates^1^, lampreys^2^, nematodes^3,4^, and various other eukaryotes^5^. A particularly remarkable example of tissue-specific genome differentiation is the germline-restricted chromosome (GRC) in the zebra finch (*Taeniopygia guttata*), which is consistently absent from somatic cells^6^. Although the zebra finch is an important animal model^7^, molecular characterisation of its GRC is limited to a short intergenic region^8^ and four genes^9,10^, rendering its evolutionary origin and functional significance largely unknown. The zebra finch GRC is the largest chromosome of this songbird^6^ and likely comprises >10% of the genome (>150 megabases)^7,11^. Cytogenetic evidence suggests that the GRC is inherited through the female germline, expelled late during spermatogenesis, and presumably eliminated from the soma during early embryonic development^6,12^. Previous analyses of a 19 kb intergenic region suggested that the GRC contains sequences with high similarity to regular chromosomes (‘A chromosomes’)^8^. Here, we combine cytogenetic, genomic, transcriptomic, and proteomic approaches to uncover the evolutionary origin and functional significance of the GRC.

## Results

### Sequencing of germline and soma genomes

In order to reliably identify sequences as GRC-linked, we used a single-molecule genome sequencing technology that permits reconstruction of long haplotypes through linked reads^13^. Haplotype phasing can aid in resolving heterozygous diploid genomes and improve the assembly of difficult genomic regions^14^. We therefore generated separate haplotype-phased de-novo genome assemblies for the germline and soma of a male zebra finch, as well as pseudohaploid versions of these assemblies (testis and liver; Seewiesen population; Supplementary Table 1). The haplotype-phased assemblies had 7.3 Mb and 0.1 Mb scaffold N50 for testis and liver, respectively, consistent with differences in input molecule lengths (Supplementary Table 1). We evaluated the performance of haplotype phasing by visually inspecting alignments of genomic regions with more than two testis haplotypes and up to two liver haplotypes (Supplementary Fig. 1a, b). This curation step validated 36 scaffolds as GRC-linked, nearly all in the range of 1–71 kb (Supplementary Fig. 1c, Supplementary Table 2). We assume that the short lengths are due to difficulties in haplotype phasing of regions where GRC and A-chromosomal haplotypes are nearly identical, i.e., regions with effectively three or more haplotypes (Supplementary Fig. 1d, e) and thus non-optimal for existing diploid assemblers. We therefore used the complementary approach of mapping linked-read data to compare testis and liver sequencing coverage and haplotype barcodes in relation to the zebra finch somatic reference genome assembly (taeGut2; generated from muscle tissue of a male individual)^7^. This allowed us to identify sequences that are shared, amplified, or unique to the germline genome, similarly to recent studies on cancer aneuploidies^15^. We also re-sequenced the germline and soma from two additional unrelated male zebra finches (Spain population; testis and muscle; Supplementary Fig. 2) using conventional PCR-free Illumina libraries as independent replicates.

### Repeat and gene content of the GRC

**Fig. 1:**
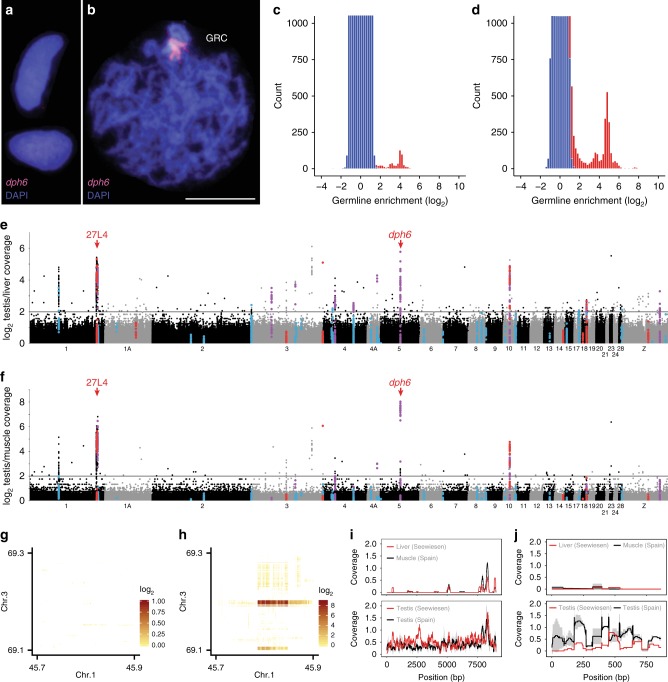
The zebra finch germline-restricted chromosome contains genes copied from many A chromosomes. **a**, **b** Cytogenetic evidence for GRC absence in muscle **a** and GRC presence in the testis **b** of the same bird (Spain_1) using fluorescence *in-situ* hybridisation (FISH) of our new GRC-amplified probe *dph6* (selected due its high germline/soma coverage ratio; *cf*. **e**, **f**). Note that the single-copy A-chromosomal paralog of *dph6* yields no visible FISH signal, unlike the estimated 308 *dph6* copies on the GRC. The scale bar indicates 10 μm. **c**, **d** Comparison of germline/soma coverage ratios for 1 kb windows with an expected symmetrical distribution (blue bars) indicates enrichment of A-chromosomal single-copy regions in the germline (red bars), similar to lamprey^2^, both in Seewiesen (**c**; linked reads) and Spain (**d**; average of Spain_1 and Spain_2 coverage; PCR-free short reads) samples. Y-axis is truncated for visualisation. **e**, **f** Manhattan plot of germline/soma coverage ratios in 1 kb windows across chromosomes of the somatic reference genome taeGut2. Colours indicate high-confidence GRC-linked genes and their identification (red: coverage, blue: SNVs, purple: both; Supplementary Table 5). Note that the similarities between Seewiesen **e** and Spain_1/Spain_2 averages **f** constitute independent biological replicates for GRC-amplified regions, as the data are based on different domesticated populations and different library preparation methods. Red arrows denote two FISH-verified GRC-amplified regions (*cf*. **b**)^8^. Only chromosomes >5 Mb are shown for clarity. **g**, **h** Linked-read barcode interaction heatmaps of an inter-chromosomal rearrangement on the GRC absent in Seewiesen liver **g** but present in Seewiesen testis **h**. **i**, **j** Coverage plots of two examples of GRC-linked genes that are divergent from their A-chromosomal paralog, *trim71* **i** and *napa* **j**^10^, and thus have very low coverage (normalised by total reads and genome size) in soma.

We first established the presence of the GRC in the three independent testis samples. Cytogenetic analysis using fluorescence in situ hybridisation (FISH) with a GRC-amplified probe (*dph6*) showed that the GRC is present exclusively in the germline and eliminated during spermatogenesis as expected (Fig. 1a, b, Supplementary Fig. 3)^6,12^. To determine whether GRC-linked sequences might stem from regular A chromosomes (i.e., autosomes or sex chromosomes), we compared germline and soma sequencing coverage by mapping reads from all three sampled zebra finches onto the somatic reference genome assembly (regular A chromosomes), revealing consistently germline-increased coverage for single-copy regions, reminiscent of programmed DNA elimination of short genome fragments in lampreys^2^ (Fig. 1c, d). A total of 92 regions (41 with >10 kb length) on 13 chromosomes exhibit >4-fold increased germline coverage relative to the soma in the Seewiesen bird (Fig. 1e, Supplementary Table 3). Such a conservative coverage cut-off provides high confidence in true GRC-amplified regions. We obtained nearly identical confirmatory results using the PCR-free library preparation for the Spain birds (Fig. 1f). Notably, the largest block of testis-increased coverage spans nearly 1 Mb on chromosome 1 and overlaps with the previously^8^ FISH-verified intergenic region 27L4 (Fig. 1e, f).

Our linked-read and re-sequencing approach allowed us to determine the sequence content of the GRC. As the GRC recombines only with itself after duplication, probably to ensure its inheritance during female meiosis^8^, it is effectively presumed to be a non-recombining chromosome. Thus, we predicted that the GRC would be highly enriched in repetitive elements, similar to the female-specific avian W chromosome (repeat density >50%, compared to <10% genome-wide)^16^. Surprisingly, neither assembly-based nor read-based repeat quantifications detected a significant enrichment in transposable elements or satellite repeats in germline samples relative to soma samples (Supplementary Fig. 4, Supplementary Table 4). Instead, most germline coverage peaks lie in single-copy regions of the reference genome overlapping with 38 genes (Fig. 1e, f, Supplementary Fig. 5, Supplementary Tables 5 and 6), suggesting that these peaks stem from very similar GRC-amplified paralogs with high copy numbers (up to 308 copies per gene; Supplementary Table 7). GRC linkage of these regions is further supported by sharing of linked-read barcodes between different amplified chromosomal regions in germline but not soma (Fig. 1g, h), suggesting that these regions reside on the same haplotype (Supplementary Fig. 6). We additionally identified 245 GRC-linked genes through germline-specific single-nucleotide variants (SNVs) present in read mapping of all three germline samples onto zebra finch reference genes (up to 402 SNVs per gene; Supplementary Table 6). As a negative control of our bioinformatic approach, we used the same methodology to screen for soma-specific SNVs and found none. We conservatively consider the 38 GRC-amplified genes and those among the 245 genes with at least 5 germline-specific SNVs as our highest-confidence set (Supplementary Table 5). We also identified GRC-linked genes using germline–soma assembly subtraction (Fig. 1i); however, all were already found via coverage or SNV evidence (Supplementary Table 5). Together with the *napa* gene recently identified in transcriptomes (Fig. 1j)^10^, our complementary approaches yielded 115 high-confidence GRC-linked genes, all of these with paralogs located on A chromosomes, i.e., 18 autosomes and the Z chromosome (Supplementary Table 5; all 267 GRC genes in Supplementary Table 6).

### Gene expression and long-term evolution of the GRC

**Fig. 2:**
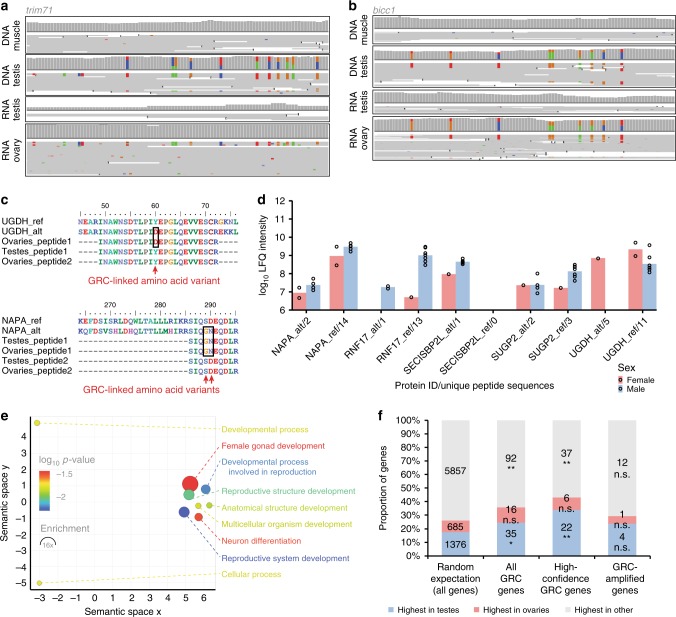
The zebra finch germline-restricted chromosome is expressed in male and female gonads. **a**, **b** Comparison of coverage and read pileups for DNA-seq data from Spain_1 and Spain_2 testis/muscle, RNA-seq data from Spain_1 and Spain_2 testis, and available ovary RNA-seq data^10^. Shown are 100-bp regions within *trim71* **a** and *bicc1* **b**. Colours indicate SNVs deviating from the reference genome taeGut2 (adenine: green; cytosine: blue; guanine: brown; thymine/uracil: red). **c** Example alignments of proteomics data showing a subset of peptide expression of the respective GRC-linked paralog of *ugdh* and *napa* (alternative or ‘alt’ peptide; *cf*. reference or ‘ref’ peptide). **d** Proteomic evidence for GRC peptide expression (‘alt’) in comparison to their A-chromosomal paralog (‘ref’) of 5 genes in 7 sampled testes and 2 sampled ovaries. For label-free quantification (LFQ), unique as well as razor (non-unique) peptides were used. Note that unique peptides may occur in several of the 9 samples. **e** Gene ontology term enrichment analysis of the 115 high-confidence GRC-linked genes (77 mapped gene symbols). Colours indicate the log_10_ of the false discovery rate-corrected *p*-value (PANTHER overrepresentation test, with a Fisher exact test for significance and filtering using a false discovery rate of 0.05), circle sizes denote fold enrichment above expected values. **f** Expression evidence for chicken orthologs of three different sets of zebra finch GRC gene paralogs in testes, ovaries, or other tissues of chicken^23^. Randomisation tests show a significant enrichment for germline-expressed genes among the chicken orthologs of 115 high-confidence GRC gene paralogs and all 267 GRC gene paralogs, but not the 38 GRC-amplified gene paralogs.

We next tested whether the GRC is physiologically functional and important, rather than facultative and purely selfish (parasitic), as presumed for supernumerary B chromosomes^17–19^, using transcriptomics and proteomics. We sequenced RNA from the same tissues of the two Spain birds used for genome re-sequencing and combined these with published testis and ovary RNA-seq data from North American domesticated zebra finches^10,20^. Among the 115 high-confidence GRC genes, we detected transcription for 6 genes in the testes and 32 in the ovaries (Supplementary Table 5). Note, these are only genes for which we could reliably separate GRC-linked and A-chromosomal paralogs using GRC-specific SNVs in the transcripts, providing an underestimate of physiologically relevant expression of the GRC (Fig. 2a, b, Supplementary Fig. 7, Supplementary Table 8). We next verified translation of GRC-linked genes through protein mass spectrometry data for 7 testes and 2 ovaries from another population (Sheffield). From 83 genes with GRC-specific amino acid changes, we identified 5 genes with peptide expression of both the paralog containing GRC-specific amino acid changes (alternative or ‘alt’), as well as the A-chromosomal paralog (reference or ‘ref’) in testes and ovaries (Fig. 2c, d, Supplementary Fig. 8, Supplementary Table 5). We therefore established that many GRC-linked genes are transcribed and translated in adult male and female gonads, extending previous RNA evidence for a single gene^10^ and rejecting the hypothesis from cytogenetic studies that the GRC is silenced in the male germline^21,22^. Instead, we propose that the GRC has important functions during germline development in both sexes, which is supported by a significant enrichment in gene ontology terms related to reproductive developmental processes among GRC-linked genes (Fig. 2e, Supplementary Table 9). We further found that the GRC is significantly enriched in genes that are also germline-expressed in GRC-lacking species (i.e., chicken^9^ and human) with RNA expression data available from many tissues^23^ (Fig. 2f, Supplementary Table 10). Specifically, out of 65 chicken orthologs of high-confidence zebra finch GRC-linked gene paralogs, 22 and 6 are most strongly expressed in chicken testis and ovary, respectively.

**Fig. 3:**
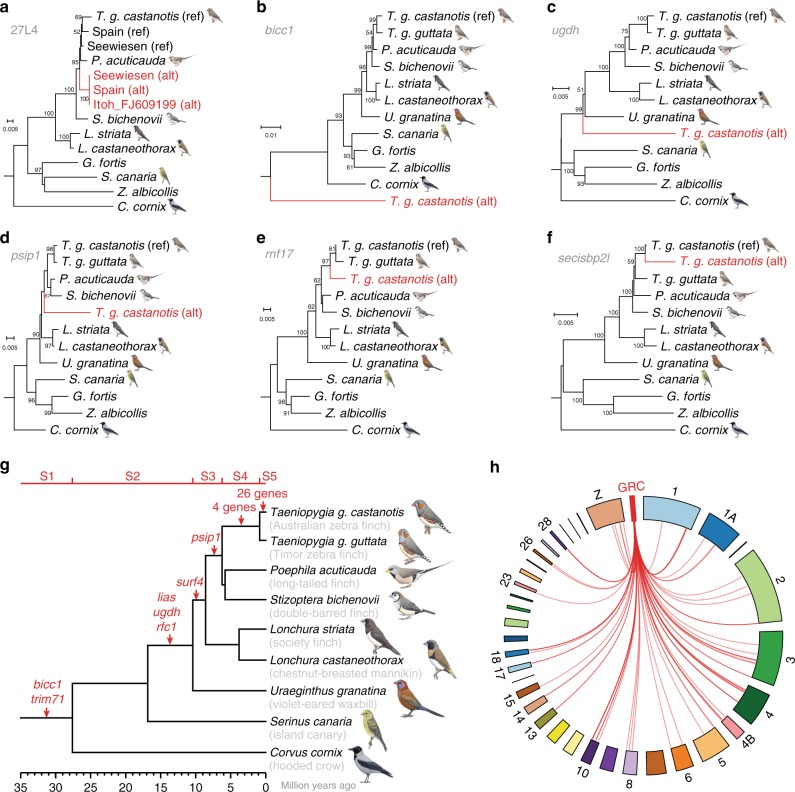
The zebra finch germline-restricted chromosome is ancient and highly dynamic. **a** Phylogeny of the intergenic 27L4 locus previously sequenced by Itoh et al.^8^ suggests stable inheritance of the GRC paralog (alternative or ‘alt’ in red; *cf*. reference or ‘ref’) among the sampled zebra finches. **b**–**f** Phylogenies of GRC-linked genes (‘alt’, in red; most selected from expressed genes) diverging from their A-chromosomal paralogs (‘ref’) before/during early songbird evolution (**b**; *bicc1*, stratum 1; *cf*. Supplementary Fig. 9), during songbird evolution (**c**; *ugdh*, stratum 2), during estrildid finch evolution (**d**; *psip1*, stratum 3), in the ancestor of the zebra finch species (**e**; *rnf17*, stratum 4), and in the Australian zebra finch subspecies (**f**; *secisbp2l*; stratum 5). The maximum likelihood phylogenies in panels **a**–**f** (only bootstrap values ≥50% shown) include available somatic genome data from estrildid finches and other songbirds. **g** Species tree of selected songbirds showing the chronological emergence of evolutionary strata (S1–S5) on the GRC (red gene names). Molecular dates are based on previous phylogenies^28,73^. Bird illustrations were used with permission from Lynx Edicions. **h** Circos plot indicating A-chromosomal origin of high-confidence GRC-linked genes from 18 autosomes and the Z chromosome. Due to the lack of chromosome-level scaffolding information for the GRC, we were unable to attribute the relative order between most of the genes in the GRC (see details in Supplementary Fig. 1c). Therefore, the represented genes are indicated in the same spot in the GRC placeholder (red box; not to scale). Note that A-chromosomal paralogs of 37 genes remain unplaced on chromosomes in the current zebra finch reference genome taeGut2.

The observation that all identified GRC-linked genes have A-chromosomal paralogs allowed us to decipher the evolutionary origins of the GRC. We utilised phylogenies of GRC-linked genes and their A-chromosomal paralogs to infer when these genes copied onto the GRC, comparable to the inference of evolutionary strata of sex chromosome differentiation^24,25^. First, the phylogeny of the intergenic 27L4 locus of our germline samples and a previous GRC sequence^8^ demonstrated stable inheritance among the sampled zebra finch populations (Fig. 3a). Second, 37 gene trees of GRC-linked genes with germline-specific SNVs and available somatic genome data from other birds identify at least five evolutionary strata (Fig. 3b–f, Supplementary Fig. 9, Supplementary Table 4), with all but stratum 3 containing expressed genes (*cf*. Fig. 2a–d). Stratum 1 emerged during early songbird diversification, stratum 2 before the diversification of estrildid finches, and stratum 3 within estrildid finches (Fig. 3g). The presence of at least 7 genes in these three strata implies that the GRC is tens of millions of years old and likely present across songbirds (Supplementary Fig. 9), consistent with a recent study reporting comprehensive cytogenetic evidence for GRC presence in all 16 songbirds analysed^9^. Notably, stratum 4 is specific to the zebra finch species and stratum 5 to the Australian zebra finch subspecies (Fig. 3g), suggesting piecemeal addition of genes from 18 autosomes and the Z chromosome over millions of years of GRC evolution (Fig. 3h). The long-term residence of expressed genes on the GRC implies that they have been under selection, such as *bicc1* and *trim71* on GRC stratum 1 whose human orthologs are important for embryonic cell differentiation^26^. Using ratios of non-synonymous to synonymous substitutions (dN/dS) for GRC-linked genes with >50 GRC-specific SNVs, we found 17 genes from all five strata evolving faster than their A-chromosomal paralogs (Supplementary Table 11). However, we also detected long-term purifying selection on 9 GRC-linked genes, including *bicc1* and *trim71*, as well as evidence for positive selection on the transcription factor *puf60*, again implying that the GRC is an important chromosome with a long evolutionary history.

## Discussion

Here we provided evidence for the origin and functional significance of a GRC. Together with recent cytogenetic evidence for GRC absence in non-passerine birds^9^, our analyses suggest that the GRC emerged during early songbird evolution. The phylogeny of the *trim71* gene (Supplementary Fig. 9a) even suggests emergence of the GRC in the common ancestor of Passeriformes, earlier than recently suggested through cytogenetic GRC presence in oscine songbirds^9,27^. Therefore, we predict the GRC to be present in half of all bird species. The species-specific addition of dozens of genes on stratum 5 implies that the rapidly evolving GRC likely contributed to reproductive isolation during the massive diversification of songbirds^28^. Previous knowledge of the gene content of the zebra finch GRC was limited to four genes (*napa*, *dph6*, *gbe1*, *robo1*)^9,10^. Our germline genome analyses expanded this gene catalogue, revealing an enrichment of germline-expressed genes on the zebra finch GRC reminiscent of nematodes and lampreys, where short genome fragments containing similar genes are eliminated during germline–soma differentiation^2–4^. All these cases constitute extreme mechanisms of gene regulation through germline–soma gene removal rather than transcriptional repression^3,5,11^. Remarkably, the GRC harbours several genes involved in the control of cell division and germline determination, including *prdm1*, a key regulator of primordial germ cell differentiation in mice^29,30^. Consequently, we hypothesise that the GRC became indispensable for its host by the acquisition of germline development genes and probably acts as a germline-determining chromosome. This might explain our evidence for RNA and protein expression of GRC genes under long-term purifying selection, and would be consistent with the previous hypothesis that GRCs are formerly parasitic B chromosomes which became stably inherited^17,18^. The aggregation of developmental genes on a single eliminated chromosome constitutes a unique mechanism to ensure germline-specific gene expression amongst multicellular organisms. Similar to what was proposed for programmed DNA elimination of short genome fragments in lamprey^31,32^, the evolution of a GRC may allow adaptation to germline-specific functions free of detrimental effects on the soma which would otherwise arise from antagonistic pleiotropy. Negative effects arising from pleiotropy of genes that are in normal circumstances active in the germline, have previously been shown in the context of cancer development^33,34^. Our results therefore have implications not only for our understanding of the function of germline-restricted DNA and the genome evolution of birds, but for how we understand resolutions to antagonistic pleiotropy, relevant to sexual conflict^35^ and the biology of disease and ageing^36^.

## Methods

### Animals and sampling

The male zebra finch (SR00100) from the Seewiesen population was part of a domesticated stock maintained at the Max Planck Institute for Ornithology in Seewiesen since 2004, a population originally derived from the University of Sheffield population described below. The specimen was four years of age when it was sacrificed and immediately dissected. Due to housing in a unisex group, it is unclear whether the male was sexually active, but at dissection its testes were of normal size (about 3–4 mm long). Testes and a sample of liver were dissected and stored in 70% ethanol before sequencing library preparation. This work complied with local laws and was carried out under the housing and breeding permit no. 311.4-si (by Landratsamt Starnberg, Germany).

The male zebra finches from the Spain population (Spain_1 and Spain_2) were bought in a pet shop in Granada. Specimens were sacrificed and dissected, extracting testes and leg muscles. Portions of testis and muscle from Spain_2 were fixed for cytogenetic study, and remaining material was immediately frozen in liquid nitrogen and stored at −80 °C before DNA and RNA extraction. This procedure was performed according to local laws and under project number 20/02/2017/027 (by Junta de Andalucía, Spain).

The zebra finches from the Sheffield population were part of a domesticated stock maintained at the University of Sheffield from 1985 to 2016. Two females and seven males were used, all aged between two and three years and reproductively active at the time of the study (i.e., females were laying eggs and males were producing sperm). The birds were maintained in breeding pairs prior to sample collection, and on the day the female laid her first egg, they were humanely euthanised by cervical dislocation under Schedule 1 (Animals (Scientific Procedures) Act 1986). Ovaries/testes were immediately dissected, washed in phosphate buffered saline solution to remove blood and connective tissue, and instantly frozen in liquid nitrogen. The entire ovary was collected from each female, as were both testes of each male. Testis and ovary samples were stored at −80 °C prior to analysis. This study was approved by the University of Sheffield, UK. All procedures performed conform to the legal requirements for animal research in the UK and were conducted under a project licence (PPL 40/3481) issued by the Home Office.

### Linked-read sequencing and genome assembly

Genomic DNA was extracted from testis and liver samples of the Seewiesen specimen using magnetic beads on a Kingfisher robot, and 10× Chromium libraries were constructed at SciLifeLab Stockholm. Libraries were multiplexed at an equimolar concentration and paired-end (2 × 150 bp) sequencing was carried out on one full lane of the Illumina HiSeq X platform (run one). For additional sequencing depth, the testis library received a further full lane of sequencing, while the liver library received a further half lane alongside a distantly related bird sample (run two). In total sequencing of testis and liver libraries generated 1,295,235,378 and 776,317,533 reads, respectively. A phased (‘megabubble’) and regular (‘pseudohaploid’) de-novo assembly was produced for each tissue from run one data (Supplementary Table 1) using Supernova^13^ v2.0. Based on these assemblies, Supernova estimated median library insert sizes of 0.28 kb and mean input molecule lengths of 62.31 kb for testis, as well as median library insert sizes of 0.31 kb and mean input molecule lengths of 28.84 kb for liver. Separately, to identify tissue-specific enrichment of sequences that were either shared between libraries or exclusive to one library, run one reads from testis and liver were compared using K-mer Analysis Toolkit^37^ v2.1.1. K-mer frequency spectra (*k* = 27) were then plotted, revealing a large enrichment of shared k-mers at high frequency in the testis, derived from repeated sequences on the GRC homologous to single-copy sequences in the soma (Supplementary Fig. 4a).

### Genome resequencing and RNA-seq

Genomic DNA was extracted from testis and leg muscle samples of the two Spain individuals using the GenElute Mammalian Genomic DNA Miniprep Kit (Sigma-Aldrich) following the manufacturer’s indications. Libraries were constructed using the Illumina TruSeq DNA PCR-Free method with an insert size of ~350 bp and sequenced on the HiSeq X Ten platform, yielding at least 17 Gb per sample (coverage ~14×) of 2 × 151 bp paired-end reads. RNA was extracted from testis and leg muscle from the same individuals using the RNeasy Lipid Tissue Kit (Qiagen) following the manufacturer’s indications. Libraries were constructed with the TruSeq mRNA Sample Prep Kit v2 and sequenced using the HiSeq4000 platform, yielding ~10 Gb per sample of 2 × 101 bp paired-end reads. Trimming was done using Trimmomatic^38^ v0.33 with options ILLUMINACLIP:TruSeq3-PE.fa:2:30:10 LEADING:3 TRAILING:3 SLIDINGWINDOW:4:20 MINLEN:100.

### Repetitive element analyses

Simple satellite repeats evolve rapidly across species and tend to accumulate on non-recombining portions of the genome^39^. The kSeek^40^ v4 pipeline was used to detect and quantify simple satellite repeats. Briefly, kSeek detects and quantifies short sequences (1–20 bp) that are tandemly repeated from unassembled reads. PCR-free reads from the Spain individuals were quality-filtered and trimmed using Trimmomatic v0.36 with options PE -phred33 ILLUMINACLIP: 2:1:10 SLIDINGWINDOW:4:20 MINLEN:20 and the k_seek.pl script was run. Quality-filtered and trimmed reads were mapped to the zebra finch somatic reference genome assembly (taeGut2; generated from muscle tissue of a male individual)^7^ using BWA-MEM^41^ v.0.7.8 with default parameters. Median insert size was obtained using the function CollectInsertSizeMetrics from Picard Tools v2.10.3. The k-mer counts were then corrected accounting for GC content using a previously published script^42^. K-mer abundance was compared for k-mers that were shared between the four samples (*n* = 257) and had a minimal count of 100. As the two samples for each tissue type were highly correlated (Pearson’s *r* > 0.98), the k-mers were averaged between samples.

To compare the number of assembled repeats in the pseudohaploid de-novo assemblies for Seewiesen liver and testis, repetitive elements were annotated using RepeatMasker^43^ v4.0.7 (‘-species Aves’). The summaries from the .tbl output files are shown in Supplementary Table 4.

To specifically detect satellite DNA, a repetitive element database was generated from taeGut2 using RepeatModeler^44^ v1.0.8. Since satellites are usually underrepresented in genome assemblies, the satMiner^45^ protocol was additionally applied to Spain testis libraries with two rounds of clustering using RepeatExplorer^46^ with 400,000 and 1,600,000 read pairs respectively. The relative genomic abundance of repeats was then compared between libraries by sampling 5 million read pairs per library and aligning them to the repeat database with RepeatMasker. A subtractive repeat landscape was generated by subtracting muscle from testis repeat abundances (Supplementary Fig. 4f).

### Cytogenetics

To demonstrate GRC presence in zebra finch germline cells and absence in somatic cells, a FISH probe to a GRC-amplified region was designed. The contigs assembled from testis libraries by RepeatExplorer as described above were clustered using CD-HIT-EST^47^, and muscle and testis reads were mapped to them using SSAHA2^48^. Two contigs with high testis versus muscle coverage ratio were selected, and were found to be homologous to an intron of the *dph6* gene (*cf*. Fig. 1e, f). Primers (Supplementary Data 1) were designed to amplify a region >500 bp from both contigs using the Primer3 software^49^. PCR amplifications were performed with initial denaturation at 95 °C for 5 min, followed by 30 cycles with 30 s denaturation at 94 °C, 30 s annealing at 60 °C, and 30 s extension at 72 °C, finishing with a final extension at 72 °C for 7 min. Cytological preparations were made from testis and leg muscle from individual Spain_2 using Meredith’s technique^50^. We labelled the *dph6* probe (Supplementary Data 1) with Tetramethylrhodamine-5-dUTP by nick translation and performed FISH in these preparations^51^. The hybridisation mix was composed of 10.5 μl formamide, 6 μl dextran sulfate, 3 μl 20×SSC, 1 μl salmon sperm, 0.5 μl SDS, 4 μl *dph6* probe, and 5 μl H_2_O, and we applied 7 min of denaturation.

Since testes contain both somatic and germline cells, the testis FISH preparations were utilised to estimate the proportion that contained a GRC. Germline cells (with FISH signal) and somatic cells (without FISH signal) were counted. The number of GRCs per haploid A genome set was calculated as 0.364, taking into account that germline cells are tetraploids and contain two GRCs, and somatic cells are diploid. A small number of polyploid cells were excluded from calculations. GRC size was estimated by measuring the relative length of synaptonemal complexes of chromosome 2 and the GRC from Figs. 1a and 2a of Pigozzi and Solari^12^. The average GRC/chromosome 2 length ratio was 1.07. Considering that chromosome 2 is 156.41 Mb in the taeGut2 reference, GRC size is estimated at 167.3 Mb. This value was used to normalise GRC copy number estimations for protein-coding genes.

### Coverage analysis

Linked reads derived from Seewiesen tissues were aligned separately to taeGut2 using Long Ranger v2.1.2 in whole genome mode. Read coverage per position of taeGut2 was calculated using the mpileup utility of Samtools^52^ v1.4. Average coverage across the genome was then calculated in windows of 1 kb and 5 kb. The smaller windows were utilised for fine-scale plotting of genome-wide coverage ratios in Fig. 1e, f, while the larger were utilised to filter GRC-amplified regions as follows. The mapping and coverage calculation was carried out again for the Spain individuals, and for the testis pseudohaploid assembly, except alignment used BWA-MEM with default settings^41^. To correct for different sequencing depth between tissues of Seewiesen, testis windows were first multiplied by the soma to testis coverage ratio. Testis windows for which the coverage was higher than the mean coverage plus two standard deviations were then removed (~5000 windows). A linear model linking window coverage in the testis as a function of the somatic sample was built, and the slope of the linear model was used to correct all testis coverage windows down (these windows were already library depth corrected). These corrections resulted in highly correlated coverage between the somatic and testis samples, with the exception of the windows that are highly amplified on the GRC (Supplementary Fig. 5a, b). The same was carried out on Spain windows, after averaging the coverage values of both individuals by tissue (Supplementary Fig. 5c, d). To filter GRC-amplified windows, the distribution of germline to soma coverage ratio was computed on a log_2_ scale. Windows with low coverage (<5^th^ percentile) in the testis sample were removed, and the distribution was centred on 0 (effectively representing a 1:1 coverage ratio between the testis and soma samples). After visual inspection of the distribution, log_2_ = 2 was selected as our coverage ratio cut-off for confident GRC-amplified windows. For Seewiesen, 510 windows were filtered and 475 for Spain, of which 465 (97.8%) were shared with Seewiesen. In all filtered windows, GC content was no lower than 30% or higher than 60%, a range where we expect read mapping not to be significantly biased. A search for somatic windows at the same excess ratio with respect to the testis returned 6 windows for Seewiesen, and none for Spain, showing the cut-off is highly conservative. Putative GRC-containing windows often occurred in blocks; Seewiesen included 51 singletons and 41 blocks of at least 10 kb, of which the largest spanned 825 kb, while Spain included 44 singletons and 36 blocks of at least 10 kb, the largest of which also spanned 825 kb.

Genes in the taeGut2 annotation of Ensembl Release 93 with overlap to putative GRC windows were identified using the intersect utility of bedtools^53^ v2.25.0, and further genes from the TransMap Ensembl V4 annotation were identified by intersection with windows via the UCSC Table Browser^54^. A total of 38 annotated genes were found.

### Structural variant analysis

Loupe outputs from Long Ranger alignment of linked-reads to taeGut2 were loaded into the 10× Genomics Loupe genome browser v2.1.1, and 11 testis-specific inter-chromosomal structural variant calls limited to anchored chromosomes were identified. No such variants were found to be liver-specific, supporting the conclusion that these represent junctions on the GRC between sequence regions with distinct A-chromosomal origins. The number of log_2_-transformed barcodes shared between structural variant coordinate ranges were plotted for the testis and liver samples using ggplot2 v3.0.0 in R v3.5.1.

### Protein-coding gene copy number analysis

Transcript sequences from the taeGut2 transcriptome were downloaded and clustered at 80% similarity across 80% of the transcript length using CD-HIT-EST set to local alignment and greedy algorithm (options -M 0 -aS 0.8 -c 0.8 -G 0 -g 1). For each tissue of the Spain and Seewiesen individuals, genomic DNA reads were then mapped to the transcriptome using SSAHA2 with an alignment score of ≥40 and minimum identity of 80%. Average read coverage per position was calculated for each transcript, normalising by library size and genome size to estimate the copy number per haploid genome with the formula: copy number = (coverage × genome size)/library size. For the genome size of somatic libraries, the taeGut2 assembly size was used (1223 Mb). For testis libraries, the size of 0.364 GRCs was added to this (yielding a total of 1329 Mb). For genes with a high variance of coverage along the sequence, regions of high coverage and regions of low coverage were split for these calculations.

### Germline DNA-specific variant analysis

Custom SNV calling was performed, selecting SNVs with ≥10 read coverage in testis but not found in somatic reads. As a negative control, the process was repeated looking for soma-specific SNVs, but none were identified. Somatic (‘ref’) and testis-specific (‘alt’) consensus sequences were generated, and coverage plots were produced using a custom script. A detailed description of this protocol can be found in Ruiz-Ruano et al.^19^ and scripts are freely available via GitHub (https://github.com/fjruizruano/whatGene). We included in our highest-confidence gene set those genes with at least 5 germline-specific SNVs (for details, see section “Gene ontology and over-representation analyses” below).

### Germline RNA-specific variant analysis

Transcription of GRC genes was demonstrated by identification of testis-specific SNVs in RNA-seq data from Spain_1, Spain_2, and published testis and ovary data^10,20^ (Supplementary Table 12). Reads were mapped to taeGut2 transcripts using SSAHA2 with options described above. Variants were identified with a minimum of 100 reads and an ‘alt’/‘ref’ ratio above 1%. Mappings were visualised using IGV^55^.

### Subtractive BLAST gene discovery

Similar to earlier work on zebra finch transcriptomes^10^, a whole-genome assembly subtractive BLAST^56^ approach was used to identify GRC-specific genes. The unmasked (so that repetitive sequences could still be identified) phased testis assembly containing 42,343 scaffolds was queried against the unmasked phased liver assembly containing 84,506 scaffolds using default BLASTn. Testis scaffolds aligning at minimum 95% identity for ≥500 bp (half the minimum scaffold length) were removed, leaving 7720. These were queried using the same algorithm and filtering options against taeGut2, leaving 3356. Next, since these scaffolds may have derived from regions difficult to assemble, the raw Sanger and 454 reads used for the taeGut2 assembly were BLASTn searched against them. Applying the same filtering criteria left 2404 scaffolds, which were then queried against an unmasked phased PacBio zebra finch genome assembly (generated from muscle tissue of the same individual as taeGut2)^14^, leaving 2020 which were regarded as ‘orphan’ scaffolds. Orphan scaffolds were queried using BLASTx against a chicken (*Gallus gallus*) SWISS-PROT database, applying an *e*-value cutoff of 1e-20 and a culling_limit of 1 (non-overlapping hits only). A total of 49 hits to 22 chicken proteins from 31 scaffolds were obtained. Pairwise MAFFT^57^ alignment was performed on the scaffolds and identical sequences were manually removed. Finally, reads from the Spain samples were mapped to the remaining scaffolds and sequences were BLASTn searched against the NCBI shotgun assembly contigs database to exclude the possibility of contamination. All scaffolds were related to bird sequences and had low coverage in the Spain library, however, all these genes had previously been identified by other approaches (specific SNVs and/or coverage).

### Mass spectrometry analysis

Testes and ovary samples were dounced and extracted using RIPA buffer (Sigma Aldrich) and quantified with the BCA Protein Quantitation Kit (Thermo Fisher Scientific). In total 150 μg total protein extract were mixed with 4× LDS sample buffer (Thermo Fisher Scientific) supplemented with 0.1 M DTT and boiled for 10 min at 70 °C prior to separation on a 12% NuPAGE Bis-Tris precast gel (Thermo Fisher Scientific) for 30 min at 170 V in MOPS buffer. The gel was fixed using the Colloidal Blue Staining Kit (Thermo Fisher Scientific) and each sample was divided into 4 equal fractions of different molecular weights. For in-gel digestion prior to MS analysis, samples were destained in destaining buffer (25 mM ammonium bicarbonate, 50% ethanol) and reduced in 10 mM DTT for 1 h at 56 °C followed by alkylation with 55 mM iodoacetamide (Sigma) for 45 min in the dark. Tryptic digest was performed in 50 mM ammonium bicarbonate buffer with 2 μg trypsin (Promega) at 37 °C overnight. Peptides were desalted on StageTips and analysed by nanoflow liquid chromatography on an EASY-nLC 1200 system coupled to a Q Exactive HF Quadrupole-Orbitrap mass spectrometer (Thermo Fisher Scientific). Peptides were separated on a C18-reversed phase column (25 cm long, 75 μm inner diameter) packed in-house with ReproSil-Pur C18-QAQ 1.9 μm resin (Dr Maisch). The column was mounted on an Easy Flex Nano Source and temperature controlled by a column oven (Sonation) at 40 °C. A 215-min gradient from 2 to 40% acetonitrile in 0.5% formic acid at a flow of 225 nl/min was used. Spray voltage was set to 2.4 kV. The Q Exactive HF was operated with a TOP20 MS/MS spectra acquisition method per MS full scan. MS scans were conducted with 60,000 at a maximum injection time of 20 ms and MS/MS scans with 15,000 resolution at a maximum injection time of 50 ms.

### Proteomic data analysis

The raw MS files were processed with MaxQuant^58^ v1.6.2.10 using the LFQ quantification^59^ option on unique peptides with at least 2 ratio counts against a single proteomic reference database generated from translated RNA-seq data of 83 high-confidence GRC-linked genes plus *napa* (‘alt’ sequences, all with at least 1 GRC-linked amino acid variant; *napa* accession MH263723.1 [https://www.ncbi.nlm.nih.gov/nuccore/MH263723.1]) and their autosomal copies (‘ref’ sequences, *napa* accession MH263724.1 [https://www.ncbi.nlm.nih.gov/nuccore/MH263724.1]), which was used to generate peptide alignments in silico. Carbamidomethylation was set as fixed modification while methionine oxidation and protein N-acetylation were considered as variable modifications. Search results were filtered with a false discovery rate of 0.01. Second peptides, dependent peptides and match between runs parameters were enabled. Both unique and razor peptides were selected for quantification. Figures were generated from the LFQ intensity data using the ggplot2 package in R.

### Gene ontology and over-representation analyses

Genes detected by all methods in addition to *napa* (*N* = 267) were compiled in a table (Supplementary Table 6) and manually curated for redundancy using NCBI, Ensembl, and UniProt lookups. In instances where multiple transcripts from the same genomic loci were identified, one gene entry was retained (X1 variant), and alternate transcripts were recorded in a separate column. We assigned genes detected in GRC-amplified regions, those with at least 5 germline-specific SNVs, and *napa* to our highest-confidence gene set (*N* = 115, Supplementary Table 5). Genomic coordinates of high-confidence genes on anchored chromosomes >5 Mb were used to annotate a Manhattan plot of testis to soma coverage ratio averaged across 1-kb windows for both the Seewiesen and Spain samples. Genes predicted to be derived from endogenous retroviruses were not plotted. A chord diagram was generated indicating the location of 81 genes from the high-confidence list that had a known location in taeGut2 using Circos^60^.

Gene symbol lists for the full and high-confidence gene sets were analysed for enrichment of gene ontology (GO) biological process terms with respect to the *Homo sapiens* reference list using the PANTHER Overrepresentation Test^61^ with a Fisher exact test for significance, and filtering of significant results using a false discovery rate of 0.05. Enriched terms were visualised using REVIGO^62^, with the SimRel semantic similarity measure and clustering at 0.9 similarity. Terms were plotted with size proportional to fold-enrichment above expected occurrence, and colour according to log_10_ of the false discovery rate *p*-value.

### Germline expression enrichment analysis

To test whether A-chromosomal paralogs of GRC-linked genes showed elevated expression levels in the gonads of species lacking a GRC, expression data across 5 male and 5 female tissues (brain, heart, kidney, liver, and gonads) were downloaded for both chicken and human^23^. Genes that were not expressed in any of the tissues were excluded. The full list of 18,616 annotated genes for taeGut2 in the Ensembl 93 release (i.e., the background zebra finch genes) was intersected with the remaining chicken and human genes. For chicken, the intersection list contained 7918 genes, of which 1376 showed their highest expression levels in testes and 685 in ovaries. Of our comprehensive list of 267 GRC-linked genes, 148 were paralogs of genes with Ensembl identities, and 143 were included in the intersection. For our high-confidence list of 115 genes (Supplementary Table 5), 75 were paralogs of genes with Ensembl identities and 65 were in the intersection. In total 17 of 36 GRC-amplified genes paralogous to genes with Ensembl identities were also in the intersection. Figure 2f shows how many chicken orthologs of the zebra finch GRC-linked gene paralogs had their highest expression in chicken testes, ovaries, or a somatic tissue. To test whether there was a higher number of genes highly expressed in the testes or ovaries than would be expected by chance, 143 (then 65, and 17) genes were randomly sampled 10,000 times from the list of 7918 genes and the number with maximal expression in the testes or ovaries were counted. For *p*-values, the fraction of the 10,000 replicates where the count for testes (or ovaries) was equal or higher than the observed count among chicken orthologs of the zebra finch GRC-linked gene paralogs is reported. Hence, these are one-tailed tests for ‘enrichment’. Likewise, one-tailed tests for ‘underrepresentation’ for the category ‘highest in other tissues’ were calculated. All observed counts and *p*-values (also for the human intersection list) are reported in Supplementary Table 10. Note that only one *p*-value survives a strict Bonferroni correction for conducting 18 hypothesis tests.

### Mitogenome analysis

The phylogenetic relationships between the zebra finch short-read libraries used in this study were calculated using the mitogenome, which was assembled for each library with a previously described whole mitogenome as a reference (haplotype A, accession DQ422742 [https://www.ncbi.nlm.nih.gov/nuccore/DQ422742])^63^ using MITObim with the quickmito protocol^64^. This protocol successfully reconstructed the mitogenome in DNA-seq and RNA-seq libraries. Assembled sequences and haplotype A were aligned with zebra finch haplotypes B-E^63^ (accessions DQ453512-15 [https://www.ncbi.nlm.nih.gov/nuccore/DQ453512,DQ453513,DQ453514,DQ453515]) with the White-rumped Munia (*Lonchura striata swinhoei*) as an outgroup^65^ (accession KR080134 [https://www.ncbi.nlm.nih.gov/nuccore/KR080134]). Alignments were with MAFFT^57^ using the ‘LINSI’ option, and uninformative sites were removed using Gblocks^66^. A phylogenetic tree was built using RAxML^67^ v8.2.12, with 100 tree searches and 100 bootstrap replicates.

### Phylogenetic analysis

GRC-linked genes may have individual evolutionary histories, so the phylogenetic relationships of each and their A-chromosomal paralogs were inferred. Since published gene transcripts from outgroup species on the NCBI Nucleotide database have different exon combinations, using these for an informative alignment would be difficult. Therefore, in addition to the soma and germline reads generated by this study, raw Sequence Read Archive reads from 10 outgroups were utilised: *Taeniopygia guttata guttata*, *Poephila acuticauda*, *Stizoptera bichenovii*, *Lonchura striata*, *Lonchura castaneothorax*, *Uraeginthus granatina*, *Serinus canaria*, *Geospiza fortis*, *Zonotrichia albicollis*, and *Corvus cornix* (Supplementary Table 12). Reads homologous to genes containing GRC-specific SNVs were filtered using BLAT^68^ with the very relaxed setting, and subsequently mapped to the same references using SSAHA2^48^ to derive a consensus sequence with the majority nucleotide for each position. Sequences with over 20% undetermined nucleotides were removed. For some genes, zebra finch testis coverage was unevenly distributed across the transcript. In these cases, only the high-coverage region was used in the alignment. In the remaining cases the whole transcript was retained. A phylogeny was built using RAxML^67^ v8.2.12 with 100 tree searches and 100 bootstrap replicates. Trees were rooted to the deepest branch among the sampled birds^69^. In the case of the genes *bicc1* and *trim71* in evolutionary stratum 1, non-passerine outgroups were included to estimate the time of their arrival on the GRC (Supplementary Fig. 9, Supplementary Tables 13 and 14). Poorly resolved trees or those that lacked sequence information from several sampled songbirds were not ranked for an evolutionary stratum. The same procedure was carried out for the previously published GRC probe 27L4 (accession FJ609199.1 [https://www.ncbi.nlm.nih.gov/nuccore/FJ609199.1]). Protocols and scripts are freely available via GitHub (https://github.com/fjruizruano/whatGene).

### Substitution rate estimation and dN/dS tests for selection

To ensure sufficient power to confidently estimate nonsynonymous to synonymous substitution rate ratios (dN/dS ratios), genes with at least 50 GRC-specific SNVs (e.g., single site substitutions) as well as *napa* were selected, including genes belonging to the five different evolutionary strata (Fig. 3g). To estimate codon specific substitution rates (dN/dS) codeml from the PAML^70^ suite v4.9 was used. Codeml input was constructed as follows. The coding parts of the DNA sequences constructed for gene phylogenetic analyses were translated into their corresponding protein sequences and prepared for codeml by backtranslating using trimAl^71^ v1.4 using the option -gt 0.2 -block 10 –splitbystopcodon. The topology from the gene tree identified in the phylogenetic analysis was used. Branch-specific models were then set up, for which a two branch type model was considered with the GRC-specific lineage as the foreground and the remaining branches as background. It was first tested whether the GRC lineage showed a significantly different dN/dS ratio compared to the rest of the tree. Secondly it was tested whether the GRC lineage shows a significantly different dN/dS ratio from 1 (indicative of neutral evolution if this hypothesis is rejected). In a third model lineage specific evidence for positive selection using the Branch-Site model A was tested. In all cases model significance was assessed with likelihood ratio tests assuming that twice the log likelihood difference is approximately χ^2^ distributed, as suggested in the PAML manual.

### Reporting summary

Further information on research design is available in the Nature Research Reporting Summary linked to this article.

## Supplementary information

Supplementary Information

Reporting Summary

Description of Additional Supplementary Files

Supplementary Data 1

Supplementary Data 2

## Data Availability

All data generated in this study have been deposited in public databases; Sequence Read Archive for the DNA and RNA sequencing data (accession numbers PRJNA552984 [https://www.ncbi.nlm.nih.gov/bioproject/PRJNA552984]), Figshare for the linked-read assemblies (10.6084/m9.figshare.8852024), and the ProteomeXchange Consortium via PRIDE^72^ for the mass spectrometry proteomics data (accession number PXD014692).

## Code availability

All custom code is freely available via GitHub (https://github.com/fjruizruano/whatGene).
