# Supplementary material for: Programmed DNA elimination of germline development genes in songbirds
Source: Nat Commun. 2019 Nov 29;10:5468. doi: 10.1038/s41467-019-13427-4 (PMC6884545; doi:10.1038/s41467-019-13427-4)
Supplement: Supplementary file 1 — Supplementary Information [file 41467_2019_13427_MOESM1_ESM.pdf]

**Supplementary Information for**

# Programmed DNA elimination of germline development genes in songbirds

**by Kinsella & Ruiz-Ruano et al.**

## Supplementary Tables

**Supplementary Table 1 | Assembly metrics of linked-read *de-novo* assemblies.** We generated assemblies from liver and testis samples of the Seewiesen zebra finch individual.

| | Liver | | Testis | |
|---|---|---|---|---|
| | **Pseudohaploid** | **Haplotype-phased** | **Pseudohaploid** | **Haplotype-phased** |
| **Number of scaffolds** | 79033 | 84506 | 40179 | 41343 |
| **Total size of scaffolds** | 1063945567 | 1451005641 | 1147364394 | 1826555761 |
| **Longest scaffold** | 2058075 | 2058075 | 86083759 | 47215586 |
| **Mean scaffold size** | 13462 | 17170 | 28556 | 44181 |
| **Median scaffold size** | 2358 | 2478 | 1824 | 1869 |
| **N50 scaffold length** | 104826 | 129001 | 17615624 | 7326433 |
| **L50 scaffold counts** | 2041 | 2618 | 17 | 49 |
| **Number of contigs** | 104187 | 117367 | 62590 | 74954 |
| **Total size of contigs** | 1027326867 | 1394498041 | 1035491694 | 1634692561 |
| **Longest contig** | 758610 | 758610 | 877267 | 706917 |
| **Mean contig size** | 9860 | 11882 | 16544 | 21809 |
| **Median contig size** | 2331 | 2723 | 2424 | 3810 |
| **N50 contig length** | 36144 | 40701 | 72584 | 75163 |
| **L50 contig count** | 7102 | 9124 | 3848 | 6060 |
| **Length of gaps (NNN)** | 36672590 | 56573560 | 111941280 | 191962940 |

**Supplementary Table 2 | List of curated GRC-linked scaffolds from haplotype-phased testis assembly.** Note that scaffold 572 likely contained a mix of GRC-linked and A-chromosomal SNVs and was therefore excluded from Supplementary Fig. 1c

| Testis phased scaffold | Gene paralog | Paralogous to A chromosome | Scaffold length | Comment |
|---|---|---|---|---|
| 352 | ZWILCH | 10 | 915868 | Several indels at 3' end |
| 572 | CENPJ | 1 | 47206955 | Scaffold excluded from Circos plot in Supplementary Fig. 1c |
| 572 | RNF17 | 1 | | |
| 727 | 27L4 | 1 | 3671 | |
| 971 | SECISBP2L | 10 | 5716 | |
| 1479 | 27L4 | 1 | 5373 | |
| 1480 | 27L4 | 1 | 5381 | |
| 1639 | VEGFA | 3 | 71033 | |
| 1639 | ELAVL4 | 8 | | Several indels at 3' end |
| 1639 | RFC1 | 4 | | |
| 1639 | WDR19 | 4 | | Indel at 3' end |
| 1640 | VEGFA | 3 | 70744 | |
| 1640 | ELAVL4 | 8 | | Several indels at 3' end |
| 1640 | RFC1 | 4 | | |
| 1640 | WDR19 | 4 | | Indel at 3' end |
| 1871 | UGDH | 4 | 4898 | |
| 1872 | UGDH | 4 | 4892 | |
| 2344 | PRPSAP1 | 18 | 20584 | |
| 50809 | 27L4 | 1 | 1111 | |
| 101007 | SUGP2 | 28 | 1203 | |
| 110465 | NRBP2 | 3 | 1484 | |
| 123035 | NRBP2 | 3 | 1095 | |
| 126612 | NAPA | Un | 1039 | |
| 140590 | UGDH | 4 | 1809 | |
| 147174 | SURF4 | 17 | 4668 | Indel at 3' end |
| 154347 | SURF4 | 17 | 7339 | Solo-LTR insertion (TguLTRK9a) |
| 158246 | BMPR1B | 4 | 8086 | |
| 158922 | UGDH | 4 | 1290 | Several indels at 3' end |
| 161446 | PUF60 | 2 | 10644 | |
| 162277 | SCRIB | 2 | 34665 | |
| 162620 | RNF17 | 1 | 3199 | |
| 164558 | SCRIB | 2 | 6769 | |
| 165075 | SHC4 | 10 | 2514 | |
| 166416 | FRS3 | 26 | 5809 | |
| 166828 | BMPR1B | 4 | 3537 | |
| 167270 | SECISBP2L | 10 | 2791 | |
| 167289 | SECISBP2L | 10 | 1427 | |
| 167469 | SECISBP2L | 10 | 1916 | |
| 167470 | SECISBP2L | 10 | 6012 | |
| 167894 | 27L4 | 1 | 1371 | |
| 168023 | 27L4 | 1 | 3477 | |
| 168469 | LIAS | 4 | 18827 | |
| 168469 | UGDH | 4 | | Several indels at 3' end |
| 169457 | SECISBP2L | 10 | 2281 | |
| 169458 | SECISBP2L | 10 | 2281 | |

**Supplementary Table 3 | Genomic blocks >10-kb with average germline/soma corrected coverage >4.** We included position with respect to the zebra finch reference genome (taeGut2), length and library source.

| Library source | Chromosome | Start | End | Length of block (kb) |
|---|---|---|---|---|
| Seewiesen | 1 | 45765001 | 45830000 | 65 |
| Spain average | 1 | 45770001 | 45795000 | 25 |
| Spain average | 1 | 45800001 | 45810000 | 10 |
| Shared by both | 1 | 104915001 | 105250000 | 335 |
| Shared by both | 1 | 105505001 | 105570000 | 65 |
| Shared by both | 1 | 105845001 | 105870000 | 25 |
| Shared by both | 1 | 105980001 | 106280000 | 300 |
| Shared by both | 1 | 106310001 | 106500000 | 190 |
| Shared by both | 1 | 106515001 | 107340000 | 825 |
| Seewiesen | 3 | 31630001 | 31645000 | 15 |
| Seewiesen | 3 | 69170001 | 69180000 | 10 |
| Shared by both | 3 | 95080001 | 95100000 | 20 |
| Seewiesen | 4 | 18975001 | 18985000 | 10 |
| Seewiesen | 4 | 48110001 | 48130000 | 20 |
| Seewiesen | 5 | 31545001 | 31590000 | 45 |
| Spain average | 5 | 31545001 | 31585000 | 40 |
| Seewiesen | 10 | 10150001 | 10180000 | 30 |
| Seewiesen | 10 | 10185001 | 10220000 | 35 |
| Spain average | 10 | 10155001 | 10180000 | 25 |
| Spain average | 10 | 10190001 | 10220000 | 30 |
| Seewiesen | 18 | 7990001 | 8020000 | 30 |
| Shared by both | 1_random | 215001 | 240000 | 25 |
| Seewiesen | 10_random | 260001 | 270000 | 10 |
| Shared by both | 3_random | 1265001 | 1280000 | 15 |
| Seewiesen | Unplaced | 149325001 | 149335000 | 10 |
| Seewiesen | Unplaced | 50480001 | 50490000 | 10 |
| Spain average | Unplaced | 127145001 | 127155000 | 10 |
| Spain average | Unplaced | 166900001 | 166910000 | 10 |
| Shared by both | Unplaced | 104835001 | 104845000 | 10 |
| Shared by both | Unplaced | 115355001 | 115365000 | 10 |
| Shared by both | Unplaced | 120935001 | 120945000 | 10 |
| Shared by both | Unplaced | 13055001 | 13065000 | 10 |
| Shared by both | Unplaced | 131635001 | 131645000 | 10 |
| Shared by both | Unplaced | 134030001 | 134040000 | 10 |
| Shared by both | Unplaced | 134420001 | 134435000 | 15 |
| Shared by both | Unplaced | 141655001 | 141665000 | 10 |
| Shared by both | Unplaced | 142710001 | 142720000 | 10 |
| Shared by both | Unplaced | 147145001 | 147155000 | 10 |
| Shared by both | Unplaced | 148110001 | 148120000 | 10 |
| Shared by both | Unplaced | 150790001 | 150800000 | 10 |
| Shared by both | Unplaced | 154710001 | 154720000 | 10 |
| Shared by both | Unplaced | 165140001 | 165150000 | 10 |
| Shared by both | Unplaced | 171530001 | 171540000 | 10 |
| Shared by both | Unplaced | 2995001 | 3005000 | 10 |

| | | | | |
|---|---|---|---|---|
| Shared by both | Unplaced | 31525001 | 31535000 | 10 |
| Shared by both | Unplaced | 32955001 | 32965000 | 10 |
| Shared by both | Unplaced | 36160001 | 36170000 | 10 |
| Shared by both | Unplaced | 66130001 | 66140000 | 10 |

**Supplementary Table 4 | Repeat annotation of the pseudohaploid testis and liver *de-novo* assemblies.** We analyzed the linked-read libraries from the Seewiesen zebra finch individual.

| | **Liver (pseudohaploid)** | | **Testis (pseudohaploid)** | | **Testis minus liver** | |
|---|---|---|---|---|---|---|
| | **copies** | **bp** | **copies** | **bp** | **copies** | **bp** |
| **SINE** | 7064 | 838534 | 7080 | 844664 | 16 | 6130 |
| **Penelope** | 109 | 21380 | 106 | 20259 | -3 | -1121 |
| **LINE** | 126192 | 36483079 | 125242 | 36875216 | -950 | 392137 |
| **LTR** | 61530 | 30202742 | 59744 | 30676032 | -1786 | 473290 |
| **DNA** | 14214 | 2192134 | 14148 | 2196181 | -66 | 4047 |
| **Rolling-circles** | 0 | 0 | 0 | 0 | 0 | 0 |
| **Unclassified** | 2398 | 421832 | 2417 | 420276 | 19 | -1556 |
| **Small RNA** | 1421 | 189079 | 1394 | 187062 | -27 | -2017 |
| **Satellites** | 674 | 64721 | 645 | 57987 | -29 | -6734 |
| **Simple repeats** | 221936 | 9618990 | 226911 | 9844941 | 4975 | 225951 |
| **Low complexity** | 44462 | 2266419 | 44990 | 2278936 | 528 | 12517 |
| **Total interspersed repeats** | 211507 | 70159701 | 208737 | 71032628 | -2770 | 872927 |
| **Total repeats** | 480000 | 82298910 | 482677 | 83401554 | 2677 | 1102644 |

**Supplementary Table 5 | Information about the 115 high-confidence genes on the GRC.** We show their A-chromosomal origin in the reference genome taeGut2, number of testis-specific SNVs, methods supporting their GRC linkage, testis/ovary RNA expression of the GRC paralog, testis/ovary protein expression of the GRC paralog, and evolutionary stratum on the GRC.

See Supplementary Data 2.

**Supplementary Table 6 | Information about all 267 genes on the GRC.** We show their A-chromosomal origin in taeGut2, number of testis-specific SNVs, methods supporting their GRC linkage, testis/ovary RNA expression of the GRC paralog, testis/ovary protein expression of the GRC paralog, and evolutionary stratum on the GRC.

See Supplementary Data 2.

**Supplementary Table 7 | Copy number estimates for 61 GRC-linked genes.** We analyzed genes with at least 2 copies on the GRC as estimated from excess coverage in testis. We consider the copy number estimates for the Spain individuals more reliable as they are based on PCR-free Illumina re-sequencing data. GRC-linked genes with low excess coverage (<0.56) in the Spain individuals are not shown and are likely present as a single copy on the GRC.

| | | | Seewiesen | | Spain | |
|---|---|---|---|---|---|---|
| **Accession** | **Annotation** | **Number of "alt" SNVs** | **Excess coverage** | **Copies on GRC** | **Excess coverage** | **Copies on GRC** |
| XM_002199579.3 | DPH6 | 13 | 92.97 | 255.4 | 112.07 | 307.9 |
| XM_012570048.1 | ROBO1 | 19 | 17.77 | 48.8 | 30.27 | 83.2 |
| XM_012577723.1 | LOC105760874 | 19 | 23.83 | 65.5 | 29.91 | 82.2 |
| XM_012570030.1 | GBE1 | 4 | 17.93 | 49.2 | 27.42 | 75.3 |
| XM_012576246.1 | LOC105759919 | 8 | 26.64 | 73.2 | 26.07 | 71.6 |
| XM_012577712.1 | LOC100222634 | 2 | 3.67 | 10.1 | 25.07 | 68.9 |
| XM_012574784.1 | LOC105759400 | 4 | 9.23 | 25.4 | 20.89 | 57.4 |
| XM_012570566.1 | LOC105758464 | 14 | 14.42 | 39.6 | 20.41 | 56.1 |
| XM_012570031.1 | LOC105758695 | 2 | 8.77 | 24.1 | 15.97 | 43.9 |
| XM_002198516.3 | BMP15 | 29 | 13.67 | 37.6 | 15.11 | 41.5 |
| XR_001123115.1 | LOC105759715 | 1 | -0.60 | -1.6 | 15.09 | 41.4 |
| XM_012575946.1 | LOC105759665 | 5 | 13.34 | 36.7 | 12.80 | 35.2 |
| XM_012575979.1 | LOC105759692 | 12 | 4.96 | 13.6 | 11.58 | 31.8 |
| XM_002197363.2 | PRPF38A | 1 | 7.18 | 19.7 | 10.69 | 29.4 |
| XM_012575943.1 | LOC105759660 | 18 | 10.82 | 29.7 | 10.29 | 28.3 |
| XM_012578155.1 | SECISBP2L | 60 | 13.50 | 37.1 | 9.71 | 26.7 |
| XM_012576350.1 | LOC105760011 | 7 | -7.47 | -20.5 | 8.56 | 23.5 |
| XM_012570047.1 | ROBO2 | 25 | 4.45 | 12.2 | 7.44 | 20.4 |
| XM_012577501.1 | LOC105760837 | 2 | 2.28 | 6.3 | 7.05 | 19.4 |
| XM_012577015.1 | LOC100228067 | 3 | -5.66 | -15.5 | 6.27 | 17.2 |
| XM_004176897.2 | COPS2 | 1 | 5.86 | 16.1 | 5.82 | 16.0 |
| XM_012576403.1 | LOC105760059 | 1 | -5.87 | -16.1 | 4.99 | 13.7 |
| XM_012576828.1 | SRRT | 16 | -1.26 | -3.5 | 4.32 | 11.9 |
| XM_012572406.1 | VEGFA | 34 | 4.31 | 11.8 | 4.00 | 11.0 |
| XM_012573358.1 | ADGRL3 | 8 | 3.62 | 9.9 | 3.94 | 10.8 |
| XM_002193695.2 | BMPR1B | 47 | 9.54 | 26.2 | 3.88 | 10.7 |
| XR_001122874.1 | LOC101233597 | 38 | 12.01 | 33.0 | 3.62 | 9.9 |
| XM_012573365.1 | UNC5C | 13 | 2.57 | 7.1 | 3.56 | 9.8 |
| XM_012576675.1 | LOC105760286 | 18 | -7.01 | -19.3 | 3.40 | 9.3 |
| XM_012576818.1 | LOC105760401 | 1 | -6.98 | -19.2 | 3.29 | 9.0 |
| XM_002186909.3 | RPRD1A | 1 | -2.29 | -6.3 | 2.91 | 8.0 |
| XM_012572672.1 | FIG4 | 17 | 0.40 | 1.1 | 2.66 | 7.3 |
| XR_001123235.1 | LOC105760076 | 1 | -5.79 | -15.9 | 2.09 | 5.7 |
| XM_002193023.2 | PSIP1 | 57 | 1.88 | 5.2 | 2.03 | 5.6 |
| XM_002188704.3 | UGDH | 136 | 3.30 | 9.1 | 2.03 | 5.6 |
| XM_012576327.1 | LOC100227204 | 3 | 0.49 | 1.4 | 1.90 | 5.2 |
| XM_002198532.3 | SHC4 | 11 | 1.96 | 5.4 | 1.72 | 4.7 |
| XM_002196665.1 | ELVAVL4 | 364 | 2.20 | 6.1 | 1.63 | 4.5 |
| XM_004174709.2 | SPHK1 | 2 | 0.39 | 1.1 | 1.32 | 3.6 |
| XM_002189420.2 | LIAS | 42 | 2.28 | 6.3 | 1.22 | 3.3 |
| XM_012571015.1 | MED20 | 28 | 1.27 | 3.5 | 1.17 | 3.2 |

| | | | | | | |
|---|---|---|---|---|---|---|
| XM_012575083.1 | LOC105759438 | 2 | -18.84 | -51.8 | 1.09 | 3.0 |
| XM_002190544.3 | RNF17 | 69 | 1.40 | 3.8 | 0.98 | 2.7 |
| XM_002186971.3 | CCND3 | 14 | 0.90 | 2.5 | 0.97 | 2.7 |
| XM_012569651.1 | CENPJ | 4 | 2.16 | 5.9 | 0.95 | 2.6 |
| XM_012573627.1 | WDR19 | 34 | 1.09 | 3.0 | 0.89 | 2.5 |
| XM_012573564.1 | RFC1 | 77 | 1.06 | 2.9 | 0.84 | 2.3 |
| XM_002195665.2 | PRPSAP1 | 7 | 1.60 | 4.4 | 0.83 | 2.3 |
| XM_012571011.1 | PGC | 24 | 0.32 | 0.9 | 0.80 | 2.2 |
| XM_012576102.1 | LOC105759795 | 3 | -3.30 | -9.1 | 0.78 | 2.1 |
| XM_012569096.1 | TTC26 | 1 | 1.08 | 3.0 | 0.77 | 2.1 |
| XM_002198572.3 | COPS2 | 1 | 2.15 | 5.9 | 0.76 | 2.1 |
| XM_012577825.1 | TIPIN | 4 | 0.57 | 1.6 | 0.75 | 2.1 |
| XM_002187632.1 | LOC100224694 | 1 | -3.11 | -8.6 | 0.75 | 2.1 |
| XM_002190902.3 | UBE3C | 1 | 2.16 | 5.9 | 0.71 | 2.0 |
| XM_012571013.1 | FRS3 | 42 | 0.73 | 2.0 | 0.69 | 1.9 |
| XM_012571795.1 | LOC105759011 | 1 | -18.06 | -49.6 | 0.68 | 1.9 |
| XM_004186121.1 | UBALD2 | 1 | 0.98 | 2.7 | 0.68 | 1.9 |
| XM_004174950.2 | ALDH18A1 | 17 | 0.65 | 1.8 | 0.59 | 1.6 |
| XM_012573167.1 | EFNB1 | 86 | 0.55 | 1.5 | 0.58 | 1.6 |
| XM_012575239.1 | ADGRL2 | 10 | 0.79 | 2.2 | 0.56 | 1.6 |

**Supplementary Table 8 | Transcriptome analyses of most expressed GRC-linked genes.** As a threshold, we consider the number of 'alt' SNVs per transcript with a minimum of 100 reads and an 'alt'/'ref' SNV mapping ratio above 1% in testes and ovary RNA-seq data. We combined the Spain_1 and Spain_2 libraries for testes and the Biederman et al.[1] library for ovary.

**Testes**

| Number of "alt" SNVs | Accession | Annotation |
|---|---|---|
| 16 | XM_012572406.1 | VEGFA |
| 14 | XM_012576828.1 | SRRT |
| 5 | XM_002187141.3 | RNF20 |
| 4 | XM_012575447.1 | LOC105759494 |
| 3 | XM_012570923.1 | PIM1 |
| 3 | XM_012576327.1 | LOC100227204 |
| 2 | XM_004174709.2 | SPHK1 |
| 1 | XM_012577445.1 | LOC101233669 |
| 1 | XM_012577723.1 | LOC105760874 |

**Ovary**

| Number of "alt" SNVs | Accession | Annotation |
|---|---|---|
| 169 | XM_012574434.1 | BICC1 |
| 60 | XM_002188704.3 | UGDH |
| 57 | XM_002189049.3 | PIM3 |
| 50 | XM_002196665.1 | ELVAVL4 |
| 45 | XM_012578155.1 | SECISBP2L |
| 37 | XR_001122874.1 | LOC101233597 |
| 34 | XM_012572406.1 | VEGFA |
| 25 | XM_002193023.2 | PSIP1 |
| 25 | XM_002198516.3 | BMP15 |
| 24 | XM_012573564.1 | RFC1 |
| 19 | XM_012573071.1 | LOC100224572 |
| 11 | XM_012572151.1 | SCRIB |
| 11 | XM_012573627.1 | WDR19 |
| 9 | XM_012575980.1 | CPEB1 |
| 8 | XM_002187141.3 | RNF20 |
| 8 | XM_012573358.1 | ADGRL3 |
| 7 | XM_002190544.3 | RNF17 |
| 7 | XM_002199183.3 | SURF4 |
| 6 | XM_002194486.2 | LOC100225333 |
| 5 | XM_012570048.1 | ROBO1 |
| 4 | NM_001245382.1 | C10H15orf61 |
| 4 | XM_012578037.1 | ZWILCH |
| 4 | XR_001123370.1 | LOC101234253 |
| 3 | XM_002188486.3 | PUF60 |
| 3 | XM_002196228.3 | AQP1 |
| 3 | XM_012578030.1 | RPL4 |
| 2 | NM_001245323.1 | AKIRIN2 |
| 2 | XM_002195665.2 | PRPSAP1 |
| 2 | XM_002196182.1 | EMG1 |

| | | |
|---|---|---|
| 2 | XM_002198098.3 | DIS3L |
| 2 | XM_012573167.1 | EFNB1 |
| 2 | XM_012576828.1 | SRRT |
| 2 | XM_012578635.1 | FEM1B |
| 1 | NM_001245147.1 | SHISAS |
| 1 | NM_001245279.2 | BCAS2 |
| 1 | NM_001245736.2 | LOC100190429 |
| 1 | XM_002193996.3 | RBBP4 |
| 1 | XM_002195155.3 | LOC100219088 |
| 1 | XM_002195523.3 | CXXC5 |
| 1 | XM_002199240.3 | LOC100220786 |
| 1 | XM_004174857.1 | PGAM1 |
| 1 | XM_004176897.2 | COPS2 |
| 1 | XM_004186121.1 | UBALD2 |
| 1 | XM_012570526.1 | LOC100218007 |
| 1 | XM_012571300.1 | DDX59 |
| 1 | XM_012571301.1 | SUGP2 |
| 1 | XM_012571388.1 | LOC100190360 |
| 1 | XM_012573977.1 | GCH1 |
| 1 | XM_012574754.1 | LOC100225911 |
| 1 | XM_012576036.1 | LOC100218716 |
| 1 | XM_012576052.1 | LOC105759754 |
| 1 | XM_012577825.1 | TIPIN |
| 1 | XM_012577979.1 | LOC100228572 |

**Supplementary Table 9 | Enriched gene ontology terms.** We show 167 mapped gene symbols from all 267 GRC-linked genes, and 77 mapped genes from 115 high confidence genes.

See Supplementary Data 2.

**Supplementary Table 10 | Enrichment analyses of orthologs of zebra finch GRC gene paralogs.** We used chicken and human RNA-seq data for testes, ovaries, and other tissues. All tests are 1-tailed, i.e., testing for enrichment (rather than underrepresentation) for genes with highest expression in gonads (testes, ovaries, either). The test for enrichment for genes with highest expression in "either testes or ovaries" is equivalent to testing for underrepresentation of genes with highest expression in "other tissues", so in Figure 2f they show up in the gray category. P-values marked with an asterisk are significant, the one marked with two asterisks survives even Bonferroni correction for doing 18 tests.

| **Expression in chicken** | **Random expectation (all genes)** | **All GRC genes** | **High-confidence GRC genes** | **GRC-amplified genes** |
|---|---|---|---|---|
| N genes | 7918 | 143 | 65 | 17 |
| highest in other tissues | 5857 | 92 | 37 | 12 |
| highest in testes | 1376 | 35 | 22 | 4 |
| highest in ovaries | 685 | 16 | 6 | 1 |
| highest in testes or ovaries | 2061 | 51 | 28 | 5 |
| highest in other tissues | 74.0% | 64.3% | 56.9% | 70.6% |
| highest in testes % | 17.4% | 24.5% | 33.8% | 23.5% |
| highest in ovaries % | 8.7% | 11.2% | 9.2% | 5.9% |
| highest in testes or ovaries % | 26.0% | 35.7% | 43.1% | 29.4% |
| p-val enriched for testes highest | | 0.019* | 0.0012** | 0.3434 |
| p-val enriched for ovaries highest | | 0.1716 | 0.4953 | 0.7782 |
| p-val enriched for t or o highest | | 0.0068* | 0.0026* | 0.4604 |
| **Expression in human** | **Random expectation (all genes)** | **All GRC genes** | **High-confidence GRC genes** | **GRC-amplified genes** |
| N genes | 8017 | 167 | 78 | 18 |
| highest in other tissues | 5879 | 121 | 59 | 16 |
| highest in testes | 1215 | 33 | 15 | 2 |
| highest in ovaries | 923 | 13 | 4 | 0 |
| highest in testes or ovaries | 2138 | 46 | 19 | 2 |
| highest in other tissues | 73.3% | 72.5% | 75.6% | 88.9% |
| highest in testes % | 15.2% | 19.8% | 19.2% | 11.1% |
| highest in ovaries % | 11.5% | 7.8% | 5.1% | 0.0% |
| highest in testes or ovaries % | 26.7% | 27.5% | 24.4% | 11.1% |
| p-val enriched for testes highest | | 0.0606 | 0.193 | 0.7804 |
| p-val enriched for ovaries highest | | 0.9564 | 0.9834 | 1 |
| p-val enriched for t or o highest | | 0.4213 | 0.7099 | 0.9734 |

**Supplementary Table 11 | Codon substitution rate analyses for 17 genes with at least 50 GRC-specific SNVs.** Omega (w = dN/dS) values denote the ratio of non-synonymous to synonymous substitution rate ratio. Likelihood differences were assessed with an LRT (likelihood ratio test statistic) assuming they are $\chi 2$ distributed. Critical values are 3.84 (5%, indicated with an asterisk) and 6.63 (1%).

| **Gene** | **Stratum** | **w (= dN/dS)** | **w (= dN/dS)** | **2xLnL difference (Branch model)** | **2xLnL difference (Branch model)** | **Positive selection in GRC** |
|---|---|---|---|---|---|---|
| | | **non-GRC branches** | **GRC branch** | **w_non-GRC = w_GRC** | **w_GRC = 1** | **Branch Site A (vs fix_w = 1)** |
| BICC1 | S1 | 0.06923 | 0.53083 | 81.75* | 14.52* | 0.78 |
| CPEB1 | | 0.05336 | 0.52899 | 56.88* | 9.14* | 3.45 |
| EFNB1 | S5 | 0.01999 | 0.30006 | 15.17* | 3.91* | 0.00 |
| ELAVL4 | | 0.03143 | 1.04632 | 77.41* | 0.03 | 0.03 |
| EPPK1 | | 0.09236 | 1.34107 | 18.45* | 0.15 | 0.14 |
| NAPA | | 0.05678 | 0.33347 | 24.49* | 11.11* | 0.41 |
| PIM1 | | 0.29867 | 0.22484 | 0.71 | 22.78* | 0.00 |
| PIM3 | | 0.02343 | 0.32847 | 14.70* | 7.84* | 0.08 |
| PSIP1 | S3 | 0.19173 | 1.00859 | 11.57* | 0.00 | 0.00 |
| PUF60 | | 0.01174 | 0.86507 | 81.31* | 0.13 | 9.38* |
| RFC1 | S2 | 0.20565 | 0.46315 | 7.78* | 6.96* | 3.82 |
| RNF17 | S4 | 0.40376 | 1.14254 | 8.66* | 0.13 | 0.87 |
| SCRIB | S5 | 0.05537 | 0.40612 | 32.35* | 8.38* | 0.02 |
| SECISBP2L | S5 | 0.05552 | 0.86945 | 25.67* | 0.07 | 0.00 |
| SURF4 | S3 | 0.07761 | 0.67209 | 11.10* | 0.57 | 0.00 |
| TRIM71 | S1 | 0.02569 | 0.32056 | 71.31* | 33.18* | 2.34 |
| UGDH | S2 | 0.01929 | 0.65357 | 54.98* | 1.27 | 0.00 |

**Supplementary Table 12 | Sequence read archive (SRA) accession numbers used for RNA-seq analyses and phylogenies.**

| Taxon | Type of material | Source | SRA accession number |
|---|---|---|---|
| *Taeniopygia guttata castanotis* | RNA ovary | Biederman et al. (2018)[1] | SRR6896649 |
| *Taeniopygia guttata castanotis* | RNA testis | Biederman et al. (2018) | SRR6896648 |
| *Taeniopygia guttata castanotis* | RNA testis | Singhal et al. (2015)[2] | SRR2299402, SRR2299403, SRR2299404 |
| *Taeniopygia guttata guttata* | RNA brain | Davidson & Balakrishnan (2016)[3] | SRR3208120, SRR3208121, SRR3208122 |
| *Poephila acuticauda* | DNA somatic | Singhal et al. (2015) | ERR1013135, ERR1013154 |
| *Stizoptera bichenovii* | DNA somatic | Singhal et al. (2015) | ERR993524 |
| *Lonchura striata domestica* | DNA somatic | Colquitt et al. (2018)[4] | SRR5223635 |
| *Lonchura castaneothorax* | DNA somatic | Stryjewski & Sorenson (2017)[5] | SRR5945143, SRR5945159, SRR5945247 |
| *Uraeginthus granatina* | RNA brain | Balakrishnan et al. (2013)[6] | SRR955502 |
| *Serinus canaria* | DNA somatic | Toomey et al. (2017)[7] | SRR2895902 |
| *Geospiza fortis* | DNA somatic | Zhang et al. (2014)[8] | SRR448675, SRR448681 |
| *Zonotrichia albicollis* | DNA somatic | Sun et al. (2018)[9] | SRR4191732 |
| *Corvus cornix* | DNA somatic | Poelstra et al. (2014)[10] | SRR862083, SRR863616, SRR863646, SRR866511, SRR866512, SRR866513, SRR866514, SRR866515, SRR866516, SRR866517, SRR866518, SRR866520, SRR866521, SRR866522, SRR866523, SRR866524 |

**Supplementary Table 13 | GenBank accession numbers used for the phylogeny of the *bicc1* gene (Supplementary Fig. 9).**

| **Species** | **Accession number(s)** |
|---|---|
| *Gallus gallus* | NM_001199507 |
| *Acanthisitta chloris* | XM_009080203 |
| *Lepidothrix coronata* | XM_017836364 |
| *Manacus vitellinus* | XM_018085397 |
| *Empidonax traillii* | PWAB01000588:702966-705855 |
| *Pseudopodoces humilis* | KB221196:1404870-1407692 |
| *Cyanistes caeruleus* | XM_023930252 |
| *Parus major* | XM_015633528 |
| *Ficedula albicollis* | CM001995:7105338-7108236 |
| *Saxicola maurus* | OAMK01000009:23946749-23949315 |
| *Passer domesticus* | CM004533:22730965-22733841 |

**Supplementary Table 14 | GenBank accession numbers used for the phylogeny of the *trim71* gene (Supplementary Fig. 9).**

| Species | Accession number(s) |
|---|---|
| *Gallus gallus* | NM_001037275 |
| *Melopsittacus undulatus* | XM_005153044 |
| *Columba livia* | XM_013370988 |
| *Falco cherrug* | XM_005445904 |
| *Acanthisitta chloris* | KK825444:47248-48614 |
| *Lepidothrix coronata* | KV388157:3238260-3239714 |
| *Manacus vitellinus* | KL669380:2291620-2292966 |
| *Empidonax traillii* | PWAB01000019:1601970-1603424 |
| *Pseudopodoces humilis* | KB221192:28949204-28950658 |
| *Cyanistes caeruleus* | PDCF01000002:8641186-8642640 |
| *Parus major* | CM003710:41203369-41204823 |
| *Ficedula albicollis* | CM001990:43120751-43122205 |
| *Saxicola maurus* | OAMK01000689:1894261-1895715 |
| *Passer domestica* | CM004529:107194472-107195926 |

## Supplementary Figures

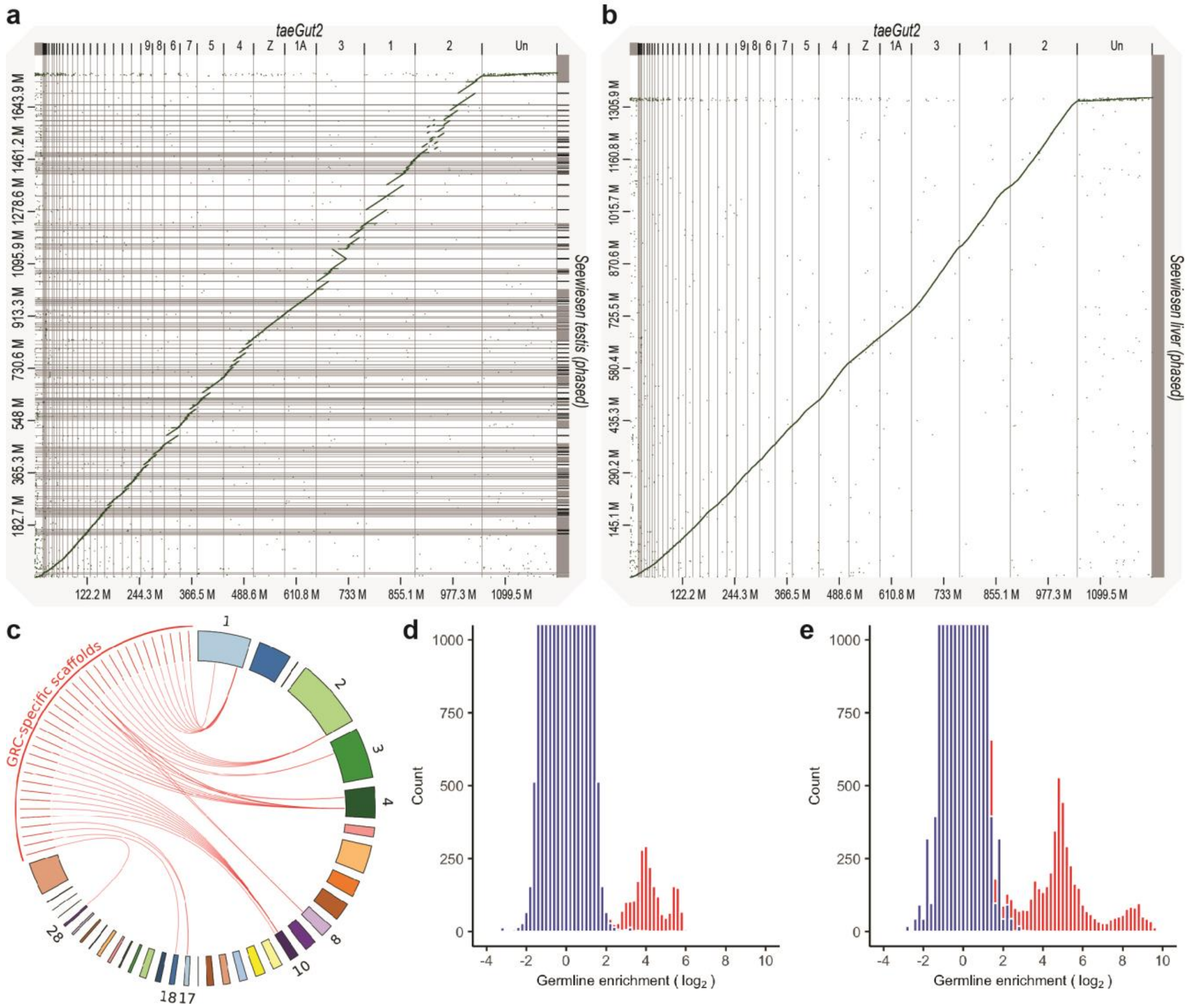


**Supplementary Figure 1 | Zebra finch germline genome assembly contains short GRC-linked scaffolds. a-b**, All-by-all dot plots of haplotype-phased testis (**a**) and liver (**b**) scaffolds (Y axis) against the somatic reference assembly taeGut2 (X axis) suggest that some regions have more than two haplotypes in testis, presumably due to difficulties in haplotype phasing of regions where GRC and A-chromosomal haplotypes are nearly identical, i.e., regions with effectively three or more haplotypes. Note that the liver assembly (**b**) is highly fragmented and individual scaffolds are not separated by horizontal lines, unlike in the testis assembly (**a**). Plots were generated with D-GENIES[11]. **c**, Circos plot of curated GRC-linked scaffolds (see scaffold names and comments in Supplementary Table 2). These selected scaffolds were manually inspected for presence of GRC-linked SNVs (identified previously via read mapping), which was necessary due to the high sequence similarity of most GRC-linked candidate regions to their A-chromosomal paralogs. This high sequence similarity likely explains the presence of both GRC-linked and A-chromosomal SNVs on one of the scaffolds (scaffold 572; i.e., probably erroneous diploid haplotype phasing in a region with effectively three or more haplotypes), which was therefore excluded from this Circos plot. Note that, although all of the verified scaffolds are short, some contain paralogs of genes from several A chromosomes (see also Supplementary Table 2). **d-e**, Comparison of germline/soma coverage ratios (red bars) for 1 kb windows with an expected symmetrical distribution (blue bars) of reads from Seewiesen (**d**) and Spain (**e**; average of Spain_1 and Spain_2 coverage) mapped against the pseudo-haploid testis assembly (cf. Figure 1c-d for the corresponding plots for reads mapped against the somatic reference assembly taeGut2).

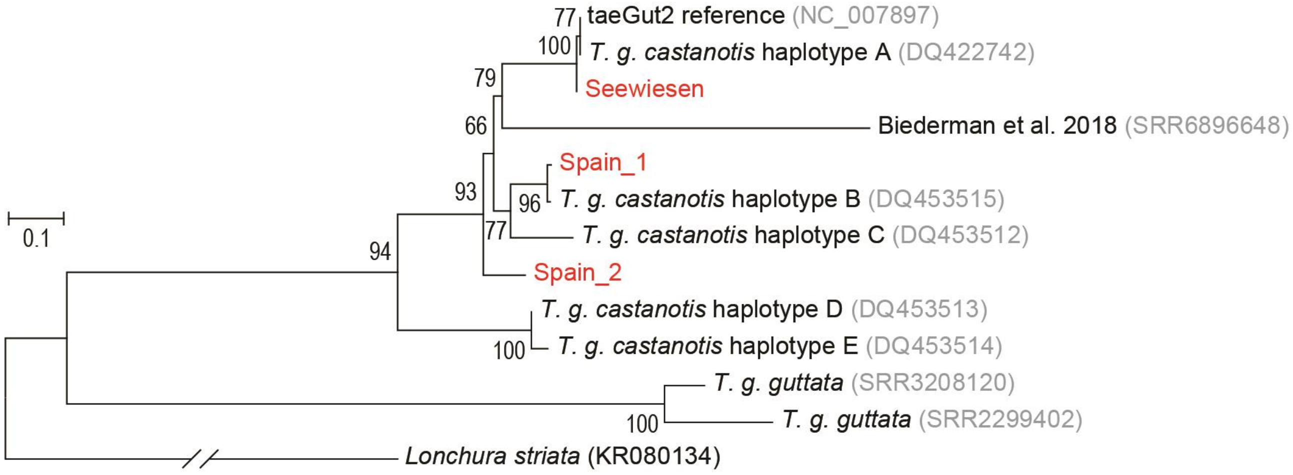


**Supplementary Figure 2 | Phylogenetic relationships between the analyzed individuals.** We built a maximum likelihood phylogeny including the five zebra finch mitochondrial haplotypes described by Mossman et al.[12] (haplotypes A-E) and mitogenomes assembled from all zebra finch Illumina libraries used in this work. These comprise both the Australian zebra finch (*Taeniopygia guttata castanotis*) and the Timor zebra finch (*Taeniopygia guttata guttata*) subspecies. Note that the three individuals sequenced by us (red colour) and by Biederman et al.[1] belong to different mitochondrial haplotypes.

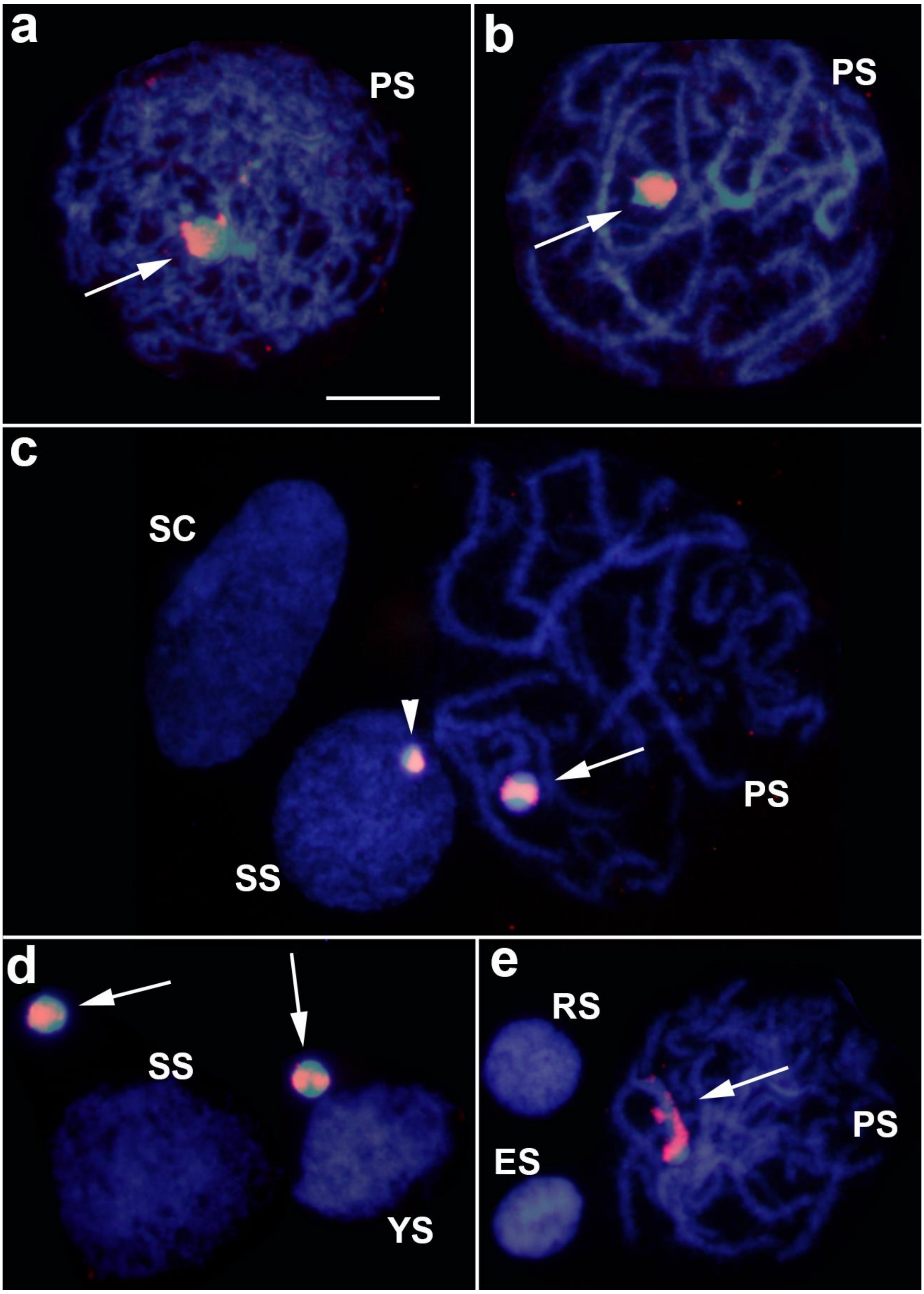


**Supplementary Figure 3 | FISH analysis in testis cells of the Spain_1 zebra finch individual.** We show the signal from the *dph6* probe in red and the counterstained with DAPI in blue. Note the presence of primary (PS) and secondary (SS) spermatocytes, young spermatids (YS) and maturing spermatids at round (RS) and elongating (ES) stages. Also note that the *dph6* probe hybridises with only part of the GRC chromosome (arrow), and this is apparent in PS at leptotene-zygotene (**a**), pachytene (**b**-**c**, **e**) and in GRCs which failed to integrate into the main nucleus of SS or YS cells (**d**), with no FISH signal in somatic cells (SC) indicating GRC absence in somatic structural testis cells (**c**). The half size of GRC in the SS cell in panel **c** (arrowhead), compared with that in the PS next to it and that those lying outside nuclei in panel **d**, suggests that GRC sometimes divides equationally in the first meiotic division (resulting in the half sized GRC body in panel **c**) but, in most cases, it divides reductionally yielding the large sized GRCs in panel **d**. Note that RS and ES nuclei in panel **e** lack FISH signal, indicating GRC absence. All photographs were made at the same magnification, and the scale bar in panel a indicates 10 μm.

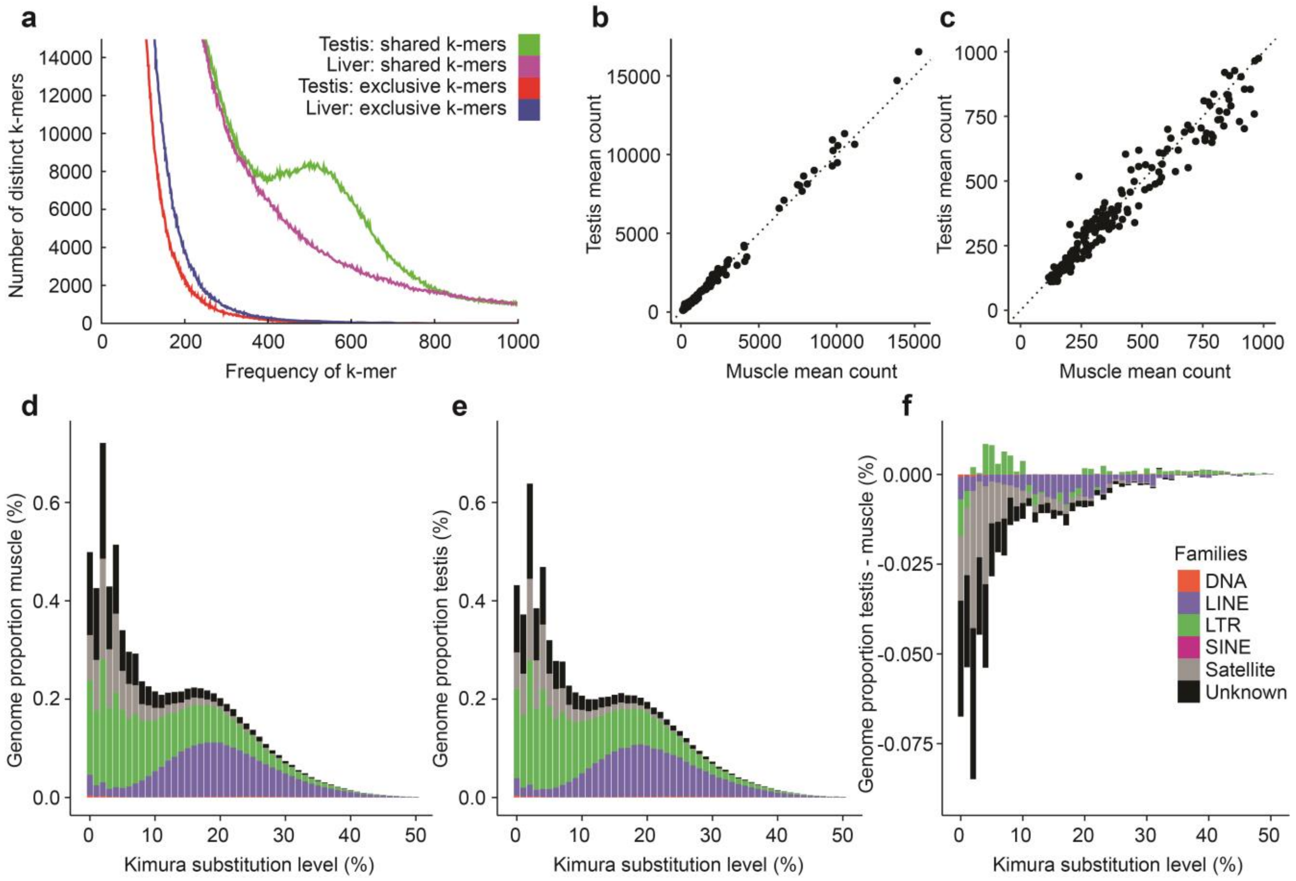


**Supplementary Figure 4 | The zebra finch GRC is not enriched in repetitive elements. a**, Comparison of spectra for k-mers shared between or exclusive to genome sequencing data from testis and liver of the Seewiesen sample, showing that the germline is not enriched for exclusive high frequency k-mers, but is conspicuously enriched in high frequency k-mers shared with the soma. **b**, Comparison of simple repeat abundance as assessed by kSeek in the Spanish muscle samples relative to the testis samples. **c**, Same as in panel b, with a focus on low abundance simple repeats. **d-e**, Repeat landscapes based on RepeatMasker analyses showing the main repetitive element families for genome re-sequencing data from muscle (**d**) and testis (**e**) of the combined Spanish samples. **f**, Subtractive repeat landscape obtained by subtracting muscle from testis counts showing a general impoverishment of testis for most of the repetitive elements (negative values) due to the presence of the GRC.

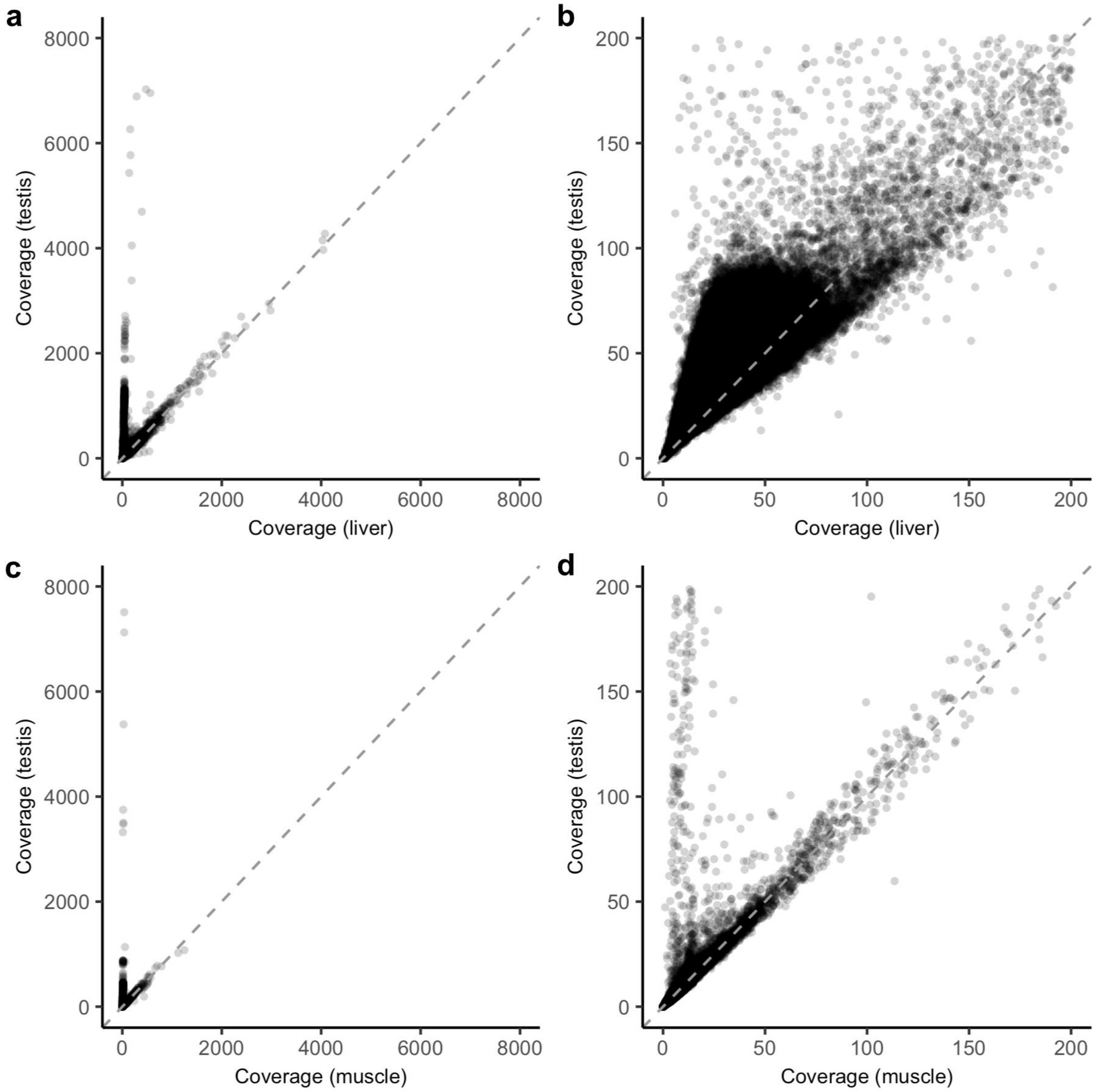


**Supplementary Figure 5 | Coverage analysis reveals highly amplified windows on the GRC.** Scatter plots showing the mean coverage of 5-kb windows in germline and soma of the Seewiesen samples (**a**; zoom-in shown in **b**) and Spain samples (**c**; zoom-in shown in **d**; average of Spain_1 and Spain_2 coverage) mapped against the somatic reference genome taeGut2. Dashed line indicates no differences between testis and muscle. Note the large number of highly amplified windows in the testis samples, revealing GRC-linked regions.

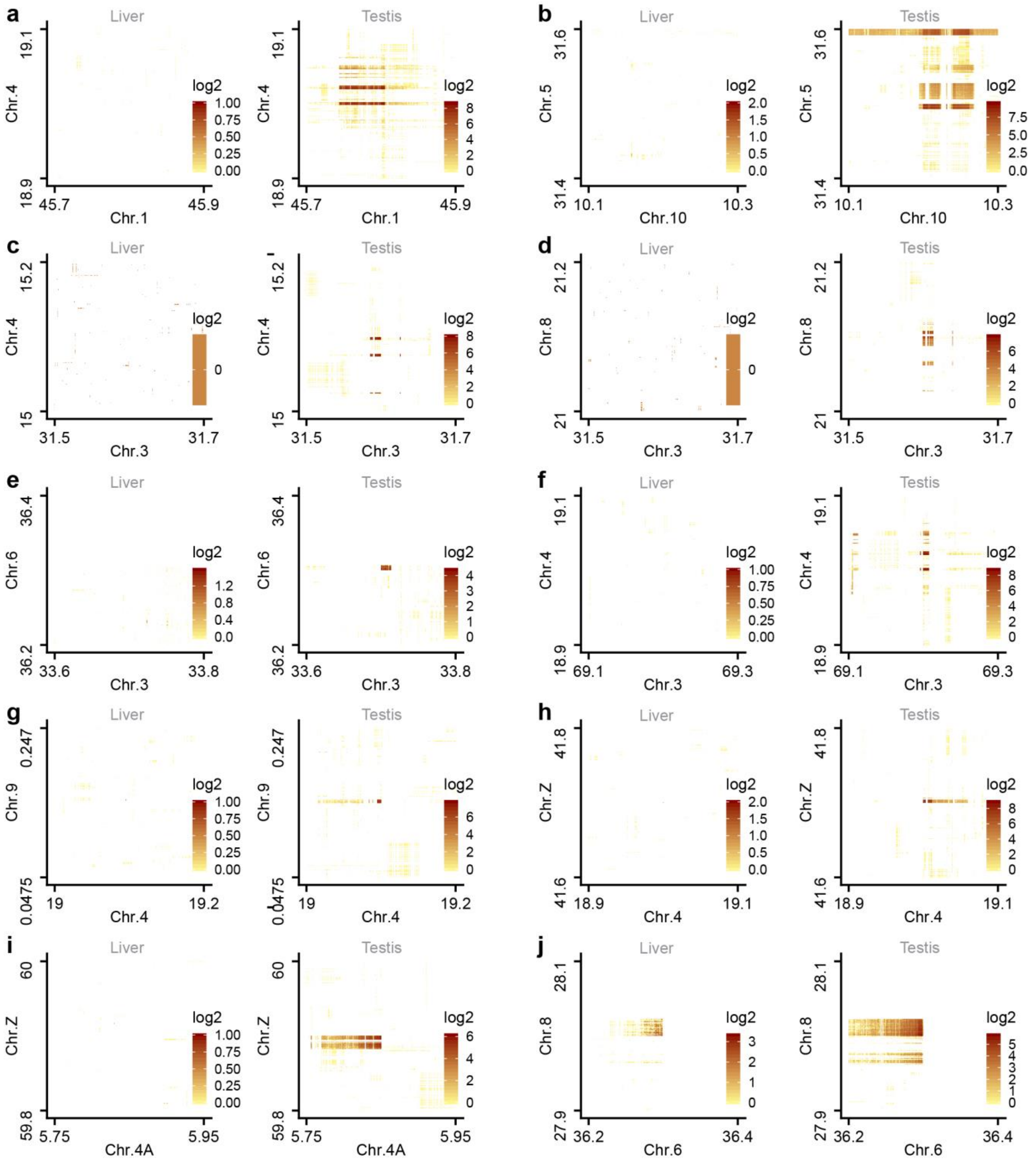


**Supplementary Figure 6 | Testis-specific linked-read barcode sharing between different A chromosomes. a-j**, Side-by-side comparison of the inter-chromosomal barcode overlap for 200-kb regions for the liver and testis, respectively (chromosome position scale in Mb). With the exception of the interaction between chromosome 6 and chromosome 8 (**j**) showing some background in the liver sample (potentially due to a shared A-chromosomal rearrangement), all inter-chromosomal structural variants were testis-specific and thus indicative of being on the same haplotype on the GRC. We exported barcode overlap matrices from the Loupe browser for testis-specific structural variants called by LongRanger and plotted them in R (v. 3.5.1). We reassigned 0 values to 'NA' (shown in white on the plot) and $\log_2$-transformed all values. Note that the scale varies across plots.

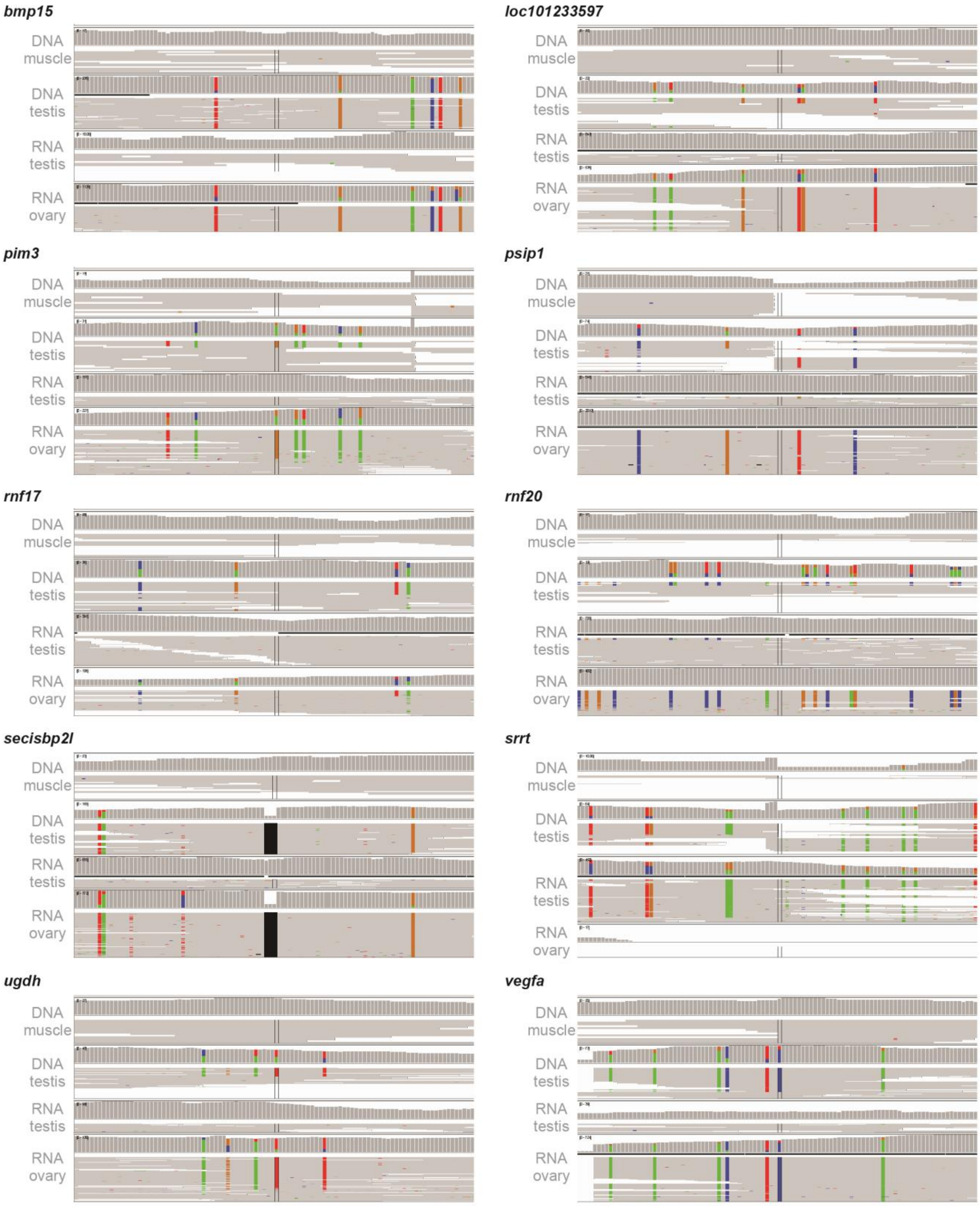


**Supplementary Figure 7 | Further examples for RNA expression of GRC-linked genes.** Comparison of coverage and read pileups for DNA-seq from Spain_1 and Spain_2 testis/muscle, RNA-seq data from Spain_1 and Spain_2 testis, and available ovary RNA-seq data[1]. Shown are 100-bp regions within 10 selected genes. Colours indicate SNVs deviating from the zebra finch reference genome taeGut2 (adenine: green; cytosine: blue; guanine: brown; thymine/uracil: red).

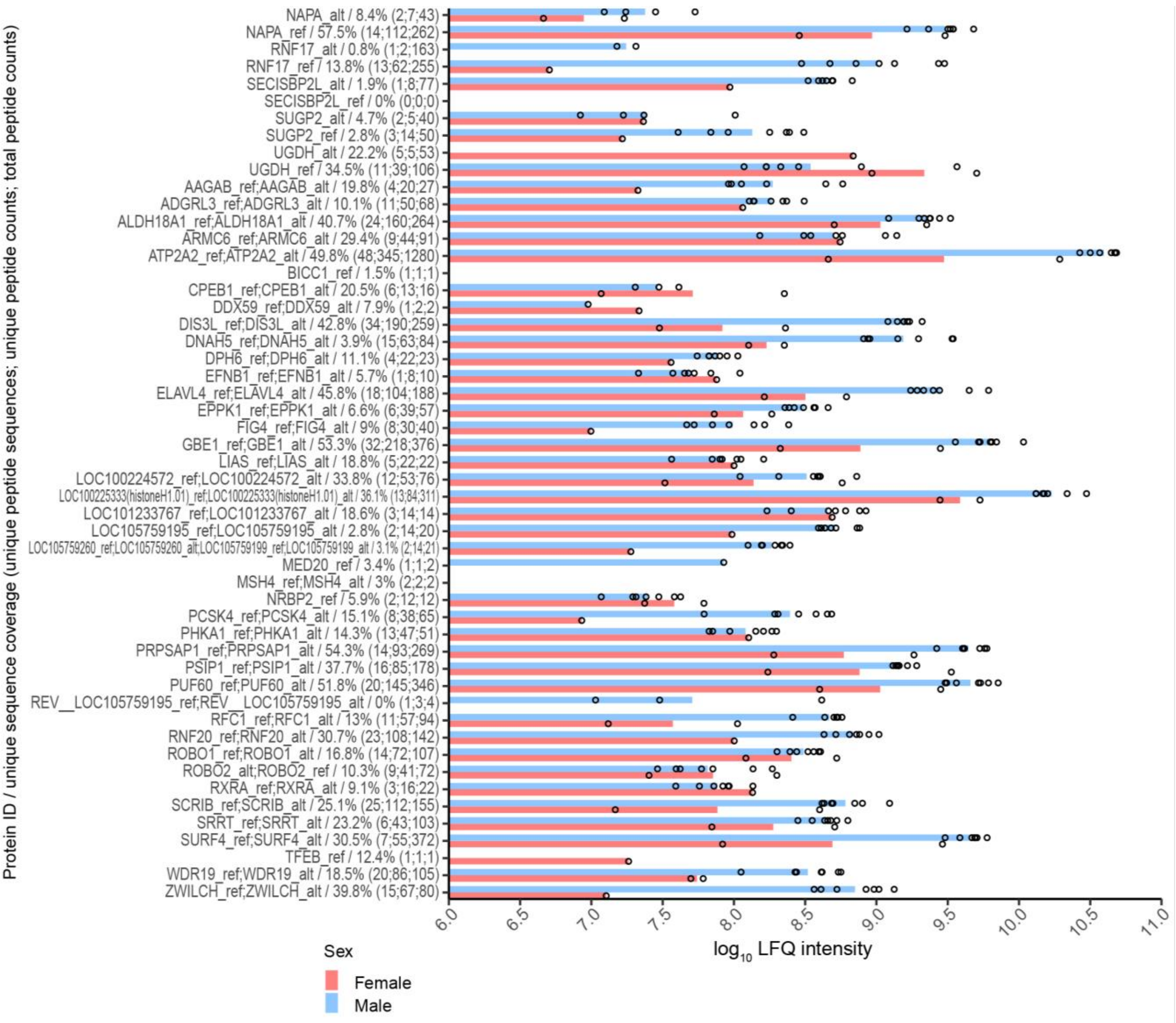


**Supplementary Figure 8 | Proteomic evidence for GRC protein presence in zebra finch testes and ovaries.** The five proteins listed at the top are also shown in Fig. 2d, i.e., those where we could differentiate between peptides from GRC vs. A chromosomes in both female (red) and male (blue) gonads. GRC paralogs are denoted by the 'alt' suffix, whereas A-chromosomal paralogs are denoted by the 'ref' suffix. Unique sequence coverage corresponds to the peptide coverage percentage of the reference protein sequence. Note that unique peptides may occur in several samples (testes/ovaries). Entries of only one protein identification have sufficient evidence at the peptide level to differentiate between the GRC and A-chromosomal paralogs due to coverage of non-identical regions between the both reference sequences; entries of more than one protein identification contain evidence of presence based solely on identical regions, thus cannot be differentiated at the proteomic level. Entries of only one protein identification without the corresponding 'alt' or 'ref' variant contain evidence that span the non-identical region only, thus the alternate variant need not be called.

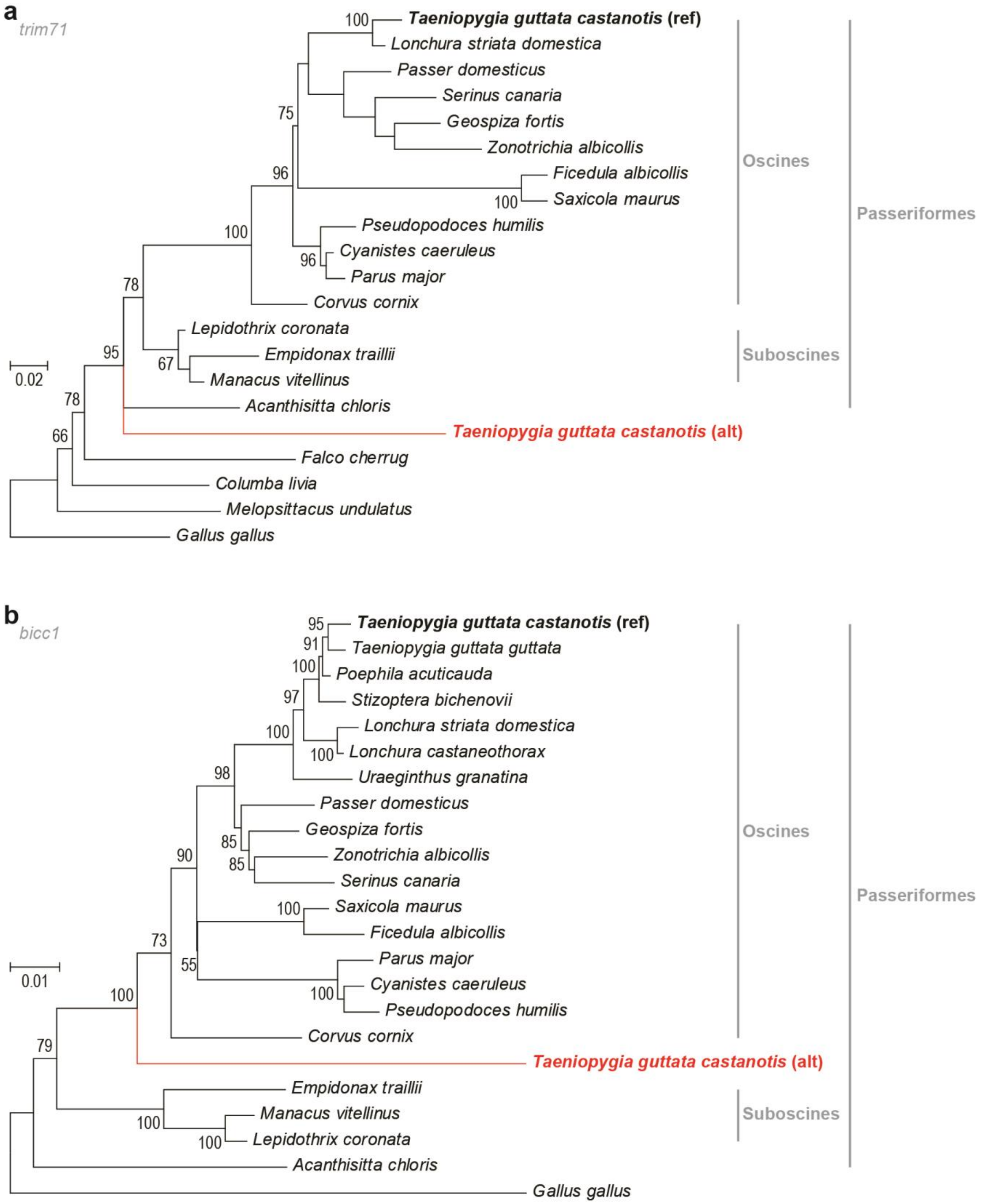


**Supplementary Figure 9 | Gene trees of GRC-linked genes from stratum 1 and their A-chromosomal paralogs.** We performed a broad taxon sampling imply GRC emergence in the ancestor of Passeriformes. Assemblied sequence from the zebra finch GRC is showed in red colour. **a**, Maximum likelihood gene tree of *trim71* (partitioned for codon positions) suggesting GRC linkage in the ancestor of Passeriformes. **b**, Maximum likelihood gene tree of *bicc1* (only 3' UTR) suggesting GRC linkage in the ancestor of oscine songbirds.

**Supplementary References**


1. Biederman, M. K. *et al.* Discovery of the first germline-restricted gene by subtractive transcriptomic analysis in the zebra finch, *Taeniopygia guttata*. *Curr. Biol.* **28**, 1620-1627.e5 (2018).
2. Singhal, S. *et al.* Stable recombination hotspots in birds. *Science* **350**, 928–932 (2015).
3. Davidson, J. H. & Balakrishnan, C. N. Gene regulatory evolution during speciation in a songbird. *G3 Genes, Genomes, Genet.* **6**, 1357–1364 (2016).
4. Colquitt, B. M., Mets, D. G. & Brainard, M. S. Draft genome assembly of the Bengalese finch, *Lonchura striata domestica*, a model for motor skill variability and learning. *GigaScience* **7**, giy008 (2018).
5. Stryjewski, K. F. & Sorenson, M. D. Mosaic genome evolution in a recent and rapid avian radiation. *Nat. Ecol. Evol.* **1**, 1912–1922 (2017).
6. Balakrishnan, C. N., Chapus, C., Brewer, M. S. & Clayton, D. F. Brain transcriptome of the violet-eared waxbill Uraeginthus granatina and recent evolution in the songbird genome. *Open Biol.* **3**, 130063 (2013).
7. Toomey, M. B. *et al.* High-density lipoprotein receptor SCARB1 is required for carotenoid coloration in birds. *Proc. Natl. Acad. Sci.* **114**, 5219–5224 (2017).
8. Zhang, G. *et al.* Comparative genomics reveals insights into avian genome evolution and adaptation. *Science* **346**, 1311–1320 (2014).
9. Sun, D., Huh, I., Zinzow-Kramer, W. M., Maney, D. L. & Yi, S. V. Rapid regulatory evolution of a nonrecombining autosome linked to divergent behavioral phenotypes. *Proc. Natl. Acad. Sci.* **115**, 2794–2799 (2018).
10. Poelstra, J. W. *et al.* The genomic landscape underlying phenotypic integrity in the face of gene flow in crows. *Science* **344**, 1410–1414 (2014).
11. Cabanettes, F. & Klopp, C. D-GENIES: dot plot large genomes in an interactive, efficient and simple way. *PeerJ* **6**, e4958 (2018).
12. Mossman, J. A., Birkhead, T. R. & Slate, J. The whole mitochondrial genome sequence of the zebra finch (*Taeniopygia guttata*). *Mol. Ecol. Notes* **6**, 1222–1227 (2006).
