# Supplementary material for: Programmed DNA elimination of germline development genes in songbirds
Source: Nat Commun. 2019 Nov 29;10:5468. doi: 10.1038/s41467-019-13427-4 (PMC6884545; doi:10.1038/s41467-019-13427-4)
Supplement: Supplementary file 2 — Reporting Summary [file 41467_2019_13427_MOESM2_ESM.pdf]

nature research

Corresponding author(s): Alexander Suh

Last updated by author(s): Nov 4, 2019

Reporting Summary

Nature Research wishes to improve the reproducibility of the work that we publish. This form provides structure for consistency and transparency in reporting. For further information on Nature Research policies, see Authors & Referees and the Editorial Policy Checklist .

Statistics

For all statistical analyses, confirm that the following items are present in the figure legend, table legend, main text, or Methods section.

n/a	Confirmed	
☐	☒	The exact sample size (n) for each experimental group/condition, given as a discrete number and unit of measurement
☐	☒	A statement on whether measurements were taken from distinct samples or whether the same sample was measured repeatedly
☐	☒	The statistical test(s) used AND whether they are one- or two-sided<br>*Only common tests should be described solely by name; describe more complex techniques in the Methods section.*
☐	☒	A description of all covariates tested
☐	☒	A description of any assumptions or corrections, such as tests of normality and adjustment for multiple comparisons
☐	☒	A full description of the statistical parameters including central tendency (e.g. means) or other basic estimates (e.g. regression coefficient) AND variation (e.g. standard deviation) or associated estimates of uncertainty (e.g. confidence intervals)
☐	☒	For null hypothesis testing, the test statistic (e.g. F, t, r) with confidence intervals, effect sizes, degrees of freedom and P value noted<br>*Give P values as exact values whenever suitable.*
☒	☐	For Bayesian analysis, information on the choice of priors and Markov chain Monte Carlo settings
☒	☐	For hierarchical and complex designs, identification of the appropriate level for tests and full reporting of outcomes
☒	☐	Estimates of effect sizes (e.g. Cohen's d, Pearson's r), indicating how they were calculated

Our web collection on statistics for biologists contains articles on many of the points above.

Software and code

Policy information about availability of computer code

Data collection

Supernova v2.0
Trimmomatic v0.33
MaxQuant v1.6.2.10

Data analysis

All custom scripts are freely available at https://github.com/fjruizruano/whatGene

K-mer Analysis Toolkit v2.1.1
kSeek v4
BWA-MEM v.0.7.8
Picard Tools v2.10.3
RepeatMasker v4.0.7
RepeatModeler v1.0.8
satMiner
RepeatExplorer
CD-HIT-EST
SSAHA2
Primer3
Long Ranger v2.1.2
Samtools v1.4
BWA v0.6.2-r126
bedtools v2.25.0
Loupe genome browser v2.1.1
ggplot2 v3.0.0

IGV
BLAST
MAFFT v7
Circos
REVIGO
MITObim
Gblocks
RAxML v8.2.12
BLAT
PAML suite v4.9

For manuscripts utilizing custom algorithms or software that are central to the research but not yet described in published literature, software must be made available to editors/reviewers. We strongly encourage code deposition in a community repository (e.g. GitHub). See the Nature Research guidelines for submitting code & software for further information.

Data

Policy information about availability of data

All manuscripts must include a data availability statement. This statement should provide the following information, where applicable:

- Accession codes, unique identifiers, or web links for publicly available datasets
- A list of figures that have associated raw data
- A description of any restrictions on data availability

All data generated in this study have been deposited in public databases; Short Read Archive for the DNA and RNA sequencing data (accession numbers PRJNA552984), Figshare for the linked-read assemblies (doi: 10.6084/m9.figshare.8852024), and the ProteomeXchange Consortium via PRIDE for the mass spectrometry proteomics data (accession number PXD014692).

Field-specific reporting

Please select the one below that is the best fit for your research. If you are not sure, read the appropriate sections before making your selection.

☒ Life sciences ☐ Behavioural & social sciences ☐ Ecological, evolutionary & environmental sciences

For a reference copy of the document with all sections, see nature.com/documents/nr-reporting-summary-flat.pdf

Life sciences study design

All studies must disclose on these points even when the disclosure is negative.

Sample size	Genome sequencing: one individual zebra finch was sequenced (liver and testis separately) from the Seewiesen population.<br>Genome re-sequencing: two zebra finches were re-sequenced from the Spain population (muscle and testis separately). Together with the Seewiesen sample, these DNA-seq data are a sufficient sample size for establishing GRC presence in genomic data.<br>RNA sequencing: the same two Spain testis samples were sequenced and complemented with publicly available RNA-seq data for testes and ovary. Together, these RNA-seq data are a sufficient sample size for establishing GRC RNA expression in adult testes.<br>Protein mass spectrometry: seven zebra finch testes and two zebra finch ovaries were sampled. Together, these proteomics data are a sufficient sample size for establishing GRC peptide expression in adult gonads.
Data exclusions	No data were excluded from the analyses.
Replication	The genome sequencing data (linked reads) and genome re-sequencing data (PCR-free short reads) constitute independent biological replicates for GRC-ampliconic regions. Testis-specific single-nucleotide variants (SNVs) were called by requiring such SNVs to be present in both the Seewiesen and Spain samples, i.e., different populations and library preparation methods.
Randomization	To test whether there was a higher number of genes highly expressed in the testes or ovaries than would be expected by chance, a set number of genes was randomly sampled 10,000 times and the number with maximal expression in the testes or ovaries counted.
Blinding	The investigators were not blinded. For testis-specific SNV calling, we used the opposite situation (soma-specific SNV calling yielding 0 such SNVs) as a negative control.

Reporting for specific materials, systems and methods

We require information from authors about some types of materials, experimental systems and methods used in many studies. Here, indicate whether each material, system or method listed is relevant to your study. If you are not sure if a list item applies to your research, read the appropriate section before selecting a response.

Materials & experimental systems

n/a	Involved in the study
☒	☐ Antibodies
☒	☐ Eukaryotic cell lines
☒	☐ Palaeontology
☐	☐ Animals and other organisms
☒	☐ Human research participants
☒	☐ Clinical data

Methods

n/a	Involved in the study
☒	☐ ChIP-seq
☒	☐ Flow cytometry
☒	☐ MRI-based neuroimaging

Animals and other organisms

Policy information about studies involving animals; ARRIVE guidelines recommended for reporting animal research

Laboratory animals	Domesticated zebra finch (Taeniopygia guttata castanotis); 1 adult male from Seewiesen population, 2 adult males from Spain population, 7 adult males and 2 adult females from Sheffield population.
Wild animals	The study did not involve wild animals.
Field-collected samples	The study did not involve samples collected from the field.
Ethics oversight	Collection of Seewiesen samples: housing and breeding permit no. 311.4-si by Landratsamt Starnberg, Germany<br>Collection of Spain samples: project number 20/02/2017/027 by Junta de Andalucía, Spain<br>Collection of Sheffield samples: project licence PPL 40/3481 issued by the Home Office, approved by the University of Sheffield, UK

Note that full information on the approval of the study protocol must also be provided in the manuscript.
