# Supplementary material for: Programmed DNA elimination of germline development genes in songbirds
Source: Nat Commun. 2019 Nov 29;10:5468. doi: 10.1038/s41467-019-13427-4 (PMC6884545; doi:10.1038/s41467-019-13427-4)
Supplement: Supplementary file 3 — Description of Additional Supplementary Files [file 41467_2019_13427_MOESM3_ESM.pdf]

Description of Additional Supplementary Files

File Name: Supplementary Data 1
Description: Sequences and primers for dph6 FISH probes.

File Name: Supplementary Data 2
Description: Contains Supplementary Table 5, Supplementary Table 6, and Supplementary Table 9.
