# Supplementary material for: Programmed DNA elimination of germline development genes in songbirds
Source: Nat Commun. 2019 Nov 29;10:5468. doi: 10.1038/s41467-019-13427-4 (PMC6884545; doi:10.1038/s41467-019-13427-4)
Supplement: Supplementary file 4 — Supplementary Data 1 [file 41467_2019_13427_MOESM4_ESM.docx]

**Supplementary Data 1 | Sequences and primers for *dph6* FISH probes.**

>tgut_seq1_r2CL30Contig1_F

GCCCAGTTGAGGGAAACTCTGGT

>tgut_seq1_r2CL30Contig1_R

ACCCTGCCTGAACTGGTCAGATG

>tgut_seq2_r2CL1Contig32_F

ACGTCTTTGCCTGACCCTTTCAGA

>tgut_seq2_r2CL1Contig32_R

TGCATAGAGTTCTCCATCAGACAGACA

>Seq1_r2CL30Contig1

TTCCAGTAATCTCCTGTGTTTTCATTAAAGAAAATCCTGGTCTTTTTCCTGTAATGATTTTAATTATCTATAGATACATGGATTAATATCTCAGAGTTGTATTTCTCAGTATTTTGGAGAAAGATTAAATTATACAAAGAGATACTTTTCTTTATGATGAATTTCCAGAATAACCACTCCTCCATAATATTTTTGCCTACATTGTAATTATTGCAAATACTGTACCCTTGATATATTGATAAGATTATCTTTGTATCTAATTCAACAGTTCAAATACAGAATTTTGTATGGCAAGTGAAATTACAGCATAAATTGAAAAAAATTGTACAAAATCGTCAATGCCCAGTTGAGGGAAACTCTGGTAGATAAAACAAAGTTATTGATCGGGCCCTTTAGTAGTTGTGTAGATTTAATGGATATTTGGAGACTACATCTCCTAGCACTGTTCTTTTTGTCTTAAAGCATGCAAGTTAAATGGGAAºTTGAAAAAATAACATTTTTTTTTTGAGAGAAAATATTTGCTGTGTTTTTTCATAGGCATGATTCCTCCACATTTTATAATGAGTAAATTTTTATCTGCAAGGATTTGAATTGTCCCTTACCCTCATTGTGTTAAATGACTACATTTTCTAATCAATATGTAATTTTTTCTTTTAATTAATTCTGGATTGGAGTTCCATAACTTCCTACATCTCGGAAATGACTGTTGTCATTAGCAGTGAATGAAATTGGGAATGAAGGGAAGAGGGTAAGAAGTAATTGCATATTTGGGAGTGATGTAAGCAGTGTAAAAATAATTATATATCTTGTTTCATTCAAACAACAGGGTTTATTTAGTTGTTGGGGTGGGCGTTGTGTGAATTTTAAAATTATATGGGAACAAGGACATTTTTTTTAATCAACAGGATGCCTGAAATTCTGTGTGTCTCATCTGACCAGTTCAGGCAGGGTCAGGATTTTAAATTTTTTTTTTTTTTTTTTTTTTTTTTTTAAGAAGGGGATCTTTGGCAAT

>Seq2_r2CL1Contig32

TCTTTAAATAAAATTGATCAACTGATTCATGAAGCCTTCTTGAATCTTCAAATATTTCTAAAATGGATTCTCTTTACTCATTTATTTCATGCTATTTTGATATGCAGTCTACCTGCATACTGCTAAAACTGAACTTCAGTTGCATTCTTCAATGTTCTTTTTAAAAAGGTTAGAAAGCTGTATGACAGTCTTGGAAAACATGACTGATGTTAAGAAGATAAATACTGTTTGTTTTTATTTTAATGCAAAAATAATAACCCTAGCAATTCCTTCAGAATGTCCTCTGTCACAATGTGCAGACAATATGTCCATAATATCAAACAATATGTCTAAAAATATTAGAGAGTTACTATATCACAAACTTAAAAAAAAAAAAATGGCACCCTCATATTAAACAAATATGGAGTGAAATCTTATTTCCCAATGACAGATTTACAGAATCTCCTGTTCATCCATGAAGATGGCAACTGACTTGCGCAATACACTTTTAACAAGCTCACAGAGCCATAAACATATTTTTTATTCATTCTGTTTTTCAAGTTGGCAAAATATATGCATCAGCAAAGATACTTGCTATGAAAACTTCCTCAATTTAAAAATAAAAGTAAATGGGAGTAGAGACTTAAATGTTATGTTCTTATACAAACACAAAAATTCAATACTAAAATGATCTATTGCACAAACTTACTTGTTTGGGATTTAAAAAATTCTAGTTGTAAGACCCAGGAAATTTCATAACCATCCTATGATAATGGAATTTTAAAATCCACAGAAGGTCACAGAATGTATACTAAATGACTATTGAAACAATGGCCCAAACTTGAAACTAATGCATCAAGAAGATTTCTCATAGGTTTTGTTTTGAGGTTGTTTGGGTGCTTTTTACTATACAAATCTATAACACATCTGATGTGCATGATTATGAATGCAAATATATATAAGTACACAAGCAAACACAAATATGTACACATATATGCAATGTATCTTGGTGTTACTAATGCAAAACCAAGGCTATTATTGGCTGCTTGCTTACAACTTAAGTTAAATTTGATTTAAATAATAACAGAGAAGAGTGTCTGCACTTATTCTAGTTTATTTCCTTTGCTAATACTTAAGTTTAAATTGTCTTTTGAAATGGCAGTACATCATGTCATTTTGCAGTTGTGGTTAACATTTATGTTTAAAAAATTCTCACAGGTATTTATTTATCGCCATCTCTTTTCATTCAGTTCTTCTCATGGTAAACAGCAAATATAAACTTTCATCACTGTCTAGACAATAGCTTCCATGAGGAGAAACACCAGTATACTTAATATTTGCTTTGAAGTGGGAGAACTAGTTAGTGAATGGGAGAACTAGTGAAATGGAATTAATTCAATCTCCCTATTTTGTCCAGTACAACAAATTAAGGCTGGAATGGCACAAATTCAAGAAATAAGATTTGAAAGGAAGAAGGATGAAGCATGAGGTAGAACAGAAAAAAAAAGCTATGGTCTAATAGCCTTCATAAACTCCTCAGCTGGACAAAGTTCACATAAAGCCTTGTCCCACGGTATATTCAGCTCAGTAGCTGAAGCATGGAAGGAACTACTAACTAATTCTAAAAAGCTAATTCTTCCCCGGACTTATTCTTTGGTATTGAAAGAGTTATTGAACACCTAGCAAACAGACAGCATTTTGTTTGACTAAGAGTTGATCATGGTTAGCAGACAATCCATCCCAGAATCAATAGTGCTGTCACTTTTAATGTGGAATCTGATCAGGTTTGGTACCATTAAAAGTGTGTTCGCAAAACTGCACTCAGCAAGGGTAAACCAAGCTGACATCTGCAGGTAAATATCAGGGATCAGCACTGAACTCAAAATCTGATGAAGTGTATTTGGGAAAATTCAGAAGGAAAGATGTTACATACTGTACCATATAAAAAGCAACAGAAAACAGAAAAAAACATCTTAGGATCCTAAGTCAATATGAAAAAGTGTTTTTTTCTTGGGTCAAGTGGATTTTTAAATTATTATTTTTAAAAGCATCATATGTTGCTAATTTAAGAGATGATTTACCTATGTTAAACTTTACTAATATGCTTCTAGTGCAATAGGCTACTCTAAAATAAAAATAAACTTCTTCTCTGGAATATTTGAATAATTCAGAAAGCTCTTATACTCAGTACAATATCCTCTGTCACAAAATATTACAACACAAAGTATGCAATGTGCACATAAAACCAGATGCTAAACACAAATGCACACAGATGCTAATGAAATTTTATTTTCCATATGACAATTTAGAACTTATTTTTCCCCTTTAGAGCTACCAAAAGTACTCATTTCAAAAGGCAGGTCGAAAAATAAACTTTTAAAAAGCAACTAAATAAGACAATTTTAGTAGCTTTTTTTTTAACGTACCGAAGATTATTATTAGCTATGTTTTAACAGTAAGTGAAATTGCACCATTCTTCTATACCTTGTTATAACAGTTAATTTATTCCTGTTCAGAGGACAAGACAATAATTTTCATAAACAATTGTGTTAGAGCAGCTAATTGATCTTTGCAACTGTAACGGTACTAAAATGCACTATTTGTGTTTAAAGTATCCTAGTTTTAAAGTTATTTATTATTTTAAACAGTATAATGAACAACAAAATTCAAAATTGCTGCTCTTTGTGTATTATAAATTACTGTTGGCTACATTTCCTATCAGGAAATAGTATAAAAAAAATAACAGTTTTGTGAAAGCTCCAAAATATCACTGCTGTTTTTAAGGTGCCTCTGATGCCTCTGTTATTTCTAAGTACACTTCTAACACACAGCTTCACAGCTAAAGTAAAAGATAAACAGATTTACACTAGCACACTGTTATTTTAATTTTGCCTTTTTTTTTTTTTCAATTAATGGTTGTAACAGTAATCAGAGGAAATTACTACATATGCATTGTAGCATATAGTACTGGTGGAGGCATTCTACCAGTAAGTAAAAAATTTATTGCAGTGGTATCAGTACAGAGGTTTTCTATAATTTCATATTTTAACGTCTTTGCCTGACCCTTTCAGAGTTCACCAAAGAGAAAATTAGTGCTAATGTTTTCAATTTAATTAGTATATTACATGCACTGTAACTATTAGTGGAAAATGTTATATTAATGAATGTAAAAGTTAGCTGTAAATTACCTCAGAGTTATTATCAATTTCCCAAGTTACTGCTCCTTAAGATAACCCTCTCCCTACGGTCCTTCCAGTAATTTTTCTTTGCCTGATAAACATGATGATTAAACAGCACACAGTGTAAACCTTTGATACTTTTTAAATTAAATCCATGCCCAGCCTAAGGGCTGTGTCTGATTTCAACTAAATGTGTCAGTTAATCAGATAAAAAATTATCAGCAACTCAAAGAAACAGCAGCCTTAGGCTTCATCAGTGCCAGAGAAAGCTGAACAAAATAAGACATACAGACAATATCACATTATGAGTAAAGGTTACTGCCAAGCATATCACTGCATAGCACTATTCACCACCACTATTATCAGTTATTTTAGAAGTTATGACAGTTTTCTCAAGTCCTTAAGGAGTCTGACACCATTGATATAGCTTTTAACACTGCATTTATGTTTATATCACAATACAATAAAACAGCAGGAATAGTGTTCAAATGACTTGGGTATTGGATGGTTTATTGAAAATAACAGTATTACCATAACATTAAAAAAATTATTTGAATGTGCTAAGTAATCACTGCTATCCATGCTGCATCTGAATTATGGGTAAATTTTGGAAGGCATCTCCAATTGCAAAGACTACTAGATCTGTCTGTCTGATGGAGAACTCTATGCATGAACAATAAATCCTAATCAAACTAAATAACCAGTAGTATGTCAATCTTGAAAAATTGTTGTGAAATCAGTGCTATGCAAATTATTTGTTCTTACAATACAGAAGACTTCATCATTACAGCTAGTATTTGCTGTTCTTTATCAGGTACAAACAAAAAGCAGAATGTAAACAACACAACCACTCTTCTTTCCATTCTTTCCCTGCAGATTGCTACATAAACCACAGTACCAATGGCACAGTACTTAATTTATCTAACTGCATTAGTAATGATTTTATCATGATTTTTTCAAAATATTTAGAGCTAACATTTTCTTACACTTGGCCTTCATTTACTCTTTGGTTAATCAAGCTCAATTCAACATCACTTGGATTTTTATAAGAAAGGAAAAGAGAGTTAATTTTTTTAAATGTCTTGATATTTTGTAGTATTTAATGCTAGAAATACAAGGCTAAATTATGTTTATCCTCTCAGACAAATTTGAAACCAGCTACATTCAAACTTGGTCAAACTTATTCACCATTTCCTAACCAAGAATTTCCATTATTTTCAATGGCAAAGAACAGCTCAGCAACACTGGTAACCAGTGATAAGGGTTTGGAGATGCACTATTACTACCACCTCAGCCCCTCCTATCAAAGGAACCTCACTTAATGAAATGCCTTGTAGAGGTTTCAAAAAGAGAAATTTGAGCTGATTTTGTATGCAGTATCACAGGCGATCACACTAGAAGTACAAGCATGGCAGGTAAAACAGTAACAATAAAAATCCATTTTCCACTGAAAGAGGATTCTTAGCCATAGACACTTCTAACCCTTCTGCCTAAGAGACTTTGCAAGTGCCCTATTAGAAAGATTAAATGGGTGCTTAGGAAAATTCATCGAAGACAGCACATACTGCAATTATGCCCAATGCATGAAGAAACAAACCCCAAAAGACAATTTGTAAAATTAGACATAGTTAGATTTATATATAATTATATATAGTGACATGTAATCACTATATGCATTTTGAAAAAAAAATAGAACCAGATATTAATCCACTGTAAAAGACTGCAGCAAAGTAAAGCAAATTAAAAATGAAGGGCCTACTGTTTTAAAATTATGTTCACTTACCCAGAATATCTATTTCAAAGCAAATGTTTGAACTCCTAATGACCTGCCTTCATGAAGTGTACATGTCTGGAACAATGACAGCTAAGAAATACTTTGCAACTTTTGCTGAGACTTGAAATACAGTCTGAATTCTACTTGAAGAGAAATCTATATAAATTTCAACATAGGCAACCACTAACTGATATGACTGCTGTTTCCTTTTCCTTTGTTGTTTTTTTTTTTTTTTTCCCTTTAATCTCTGATGCAGTCTTTTCTCCTTTTGCAT
